# Regional changes in CNS and retinal glycerophospholipid profiles with age: a molecular blueprint[S]

**DOI:** 10.1194/jlr.M070714

**Published:** 2017-03-29

**Authors:** Blake R. Hopiavuori, Martin-Paul Agbaga, Richard S. Brush, Michael T. Sullivan, William E. Sonntag, Robert E. Anderson

**Affiliations:** Oklahoma Center for Neuroscience,* University of Oklahoma Health Sciences Center, Oklahoma City, OK 73104; Department of Ophthalmology,† University of Oklahoma Health Sciences Center, Oklahoma City, OK 73104; Department of Geriatric Medicine,†† University of Oklahoma Health Sciences Center, Oklahoma City, OK 73104; Department of Cell Biology,§ University of Oklahoma Health Sciences Center, Oklahoma City, OK 73104; Dean McGee Eye Institute,** Oklahoma City, OK 73104

**Keywords:** brain lipids, brain, fatty acid, phospholipids, phospholipids/phosphatidylcholine, phospholipids/phosphatidylethanolamine, phospholipids/phosphatidylserine, plasmalogens, very long-chain polyunsaturated fatty acids, aging, central nervous system

## Abstract

We present here a quantitative molecular blueprint of the three major glycerophospholipid (GPL) classes, phosphatidylcholine (PC), phosphatidylserine (PS), and phosphatidylethanolamine (PE), in retina and six regions of the brain in C57Bl6 mice at 2, 10, and 26 months of age. We found an age-related increase in molecular species containing saturated and monoenoic FAs and an overall decrease in the longer-chain PUFA molecular species across brain regions, with loss of DHA-containing molecular species as the most consistent and dramatic finding. Although we found very-long-chain PUFAs (VLC-PUFAs) (⩾C28) in PC in the retina, no detectable levels were found in any brain region at any of the ages examined. All brain regions (except hippocampus and retina) showed a significant increase with age in PE plasmalogens. All three retina GPLs had di-PUFA molecular species (predominantly 44:12), which were most abundant in PS (∼30%). In contrast, low levels of di-PUFA GPL (1–2%) were found in all regions of the brain. This study provides a regional and age-related assessment of the brain’s lipidome with a level of detail, inclusion, and quantification that has not heretofore been published.

In the last few decades, neuroscientists have begun to identify and elucidate many unexpected and dynamic roles of lipid molecules in the brain (1). These changes include maintaining the biophysical properties of lipid rafts (2), regulating ion channel and receptor activities (3–12), protecting neurons from oxidant and other stresses (13–15), and regulating neuronal gene transcription (16–18) and neurotransmitter release (19). With more and more neuronal roles being identified each year for complex lipid molecules, FAs, and bioactive lipid derivatives, it is imperative that we have a molecular blueprint of which lipid classes are expressed in which regions of the brain and the molecular species they contain. By generating such a blueprint, we can begin a targeted approach to understanding the potentially multifaceted roles for these molecules in regulating and maintaining neuronal development, health, and function.

Since the early pioneering work of Folch-Pi (20–22), Ansell and Spanner (23–26), Cotman and coworkers (27–30), Poulos and coworkers (31–34), and Rouser and coworkers (35–40), to name a few, there have been other studies analyzing the lipid composition of the brain, and some with age; however, these were mostly focused around a specific molecule, class of molecules, or, in most cases, individual FAs. There have been a number of reports on the lipid profiles of postmortem human brains under normal aging conditions, with various levels of cognitive impairment, and those with Alzheimer’s or other dementias. The primary focus of many of these studies was on bioactive lipids such as DHA and arachidonic acid (AA), with emphasis on the relative percent composition of n3 and n6 FAs, lipid modification of age-induced alterations in gene expression, and relative percent composition of certain lipid classes (3, 4, 16, 41–43). To our knowledge, there has been no thorough comparative analysis of the age-related changes in major glycerophospholipid (GPL) profiles in multiple regions of the brain and the retina. Like any other mammalian organ system, the brain changes as a consequence of age (44–46). There is some degree of evidence that components of the lipidome have been shown to change with age and have been linked to pathological degeneration and disease (41, 42, 47–49). Yet, the age-related lipid profiles of the various brain regions have not been adequately characterized, and, thus, it is important to determine whether significant age- and region-specific changes occur and, if so, to define the nature of those alterations.

We hypothesize, first, that the various regions of the CNS are composed of unique compositions of lipid molecules that depend on that region’s function, and, second, that the relative composition of these molecules changes differentially with age. We chose the aging mouse due to its close genetic proximity to humans, its reliable use in aging research due to its relatively short life span (50), and its malleability to genetic engineering. Using significant advances in the technology associated with lipidomic analysis, we combined traditional lipid biochemistry with new and cutting-edge technology to evaluate the regional lipid composition of the mouse brain with a level of detail, scrutiny, and inclusion that to date has not been done. In the present study, we present a detailed quantitative molecular species analysis of the three major GPL classes, phosphatidylcholine (PC), phosphatidylethanolamine (PE), and phosphatidylserine (PS), in retina, hippocampus, cerebellum, brainstem, cortex, white matter, and midbrain of 2-, 10-, and 26-month-old mice.

## MATERIALS AND METHODS

### Animals

C57Bl6 mice of mixed sex and ages 2, 10, and 26 months were purchased from the National Institute on Aging (NIA) and acclimated in the University of Oklahoma Health Sciences Center (OUHSC) vivarium for at least 2 weeks (12 h ON; 12 h OFF, ∼150 lux). The animals were fed PicoLab irradiated 5053 lab diet (LabDiet®, Land O’Lakes Inc., St. Louis, MO) ad libitum. The animals were monitored routinely for endo and ecto parasites; blood samples are taken quarterly from sentinel mice as part of our health monitoring of all rodents to exclude most bacteria and viruses. Animals were euthanized by cervical dislocation followed by decapitation. The following tissues were dissected, frozen, and stored at −80°C: retina, hippocampus, cerebellum, brainstem, cortex, white matter, and midbrain.

All procedures were performed in accordance with the Association for Research in Vision and Ophthalmology Statement for the Use of Animals in Ophthalmic and Vision Research and the UOHSC Guidelines for Animals in Research. All protocols were reviewed and approved by the Institutional Animal Care and Use Committees of the UOHSC.

### Tandem MS analysis of lipids

The methods have been described previously (51). Briefly, tissue was homogenized in 40% aqueous methanol and then diluted to a concentration of 1:40 with 2-propanol/methanol/chloroform (4:2:1 v/v/vol) containing 20 mM ammonium formate and 1.0 µM PC (14:0/14:0), 1.0 µM PE (14:0/14:0), 0.33 µM PS (14:0/14:0), and 12.5 nM ceramide (d18:1/12:0) as internal standards. Samples were introduced into a triple-quadrupole mass spectrometer (TSQ Ultra, Thermo Scientific, Oakwood Village, OH) by using a chip-based nano-ESI source (Advion NanoMate, Advion, Ithaca, NY) operating in infusion mode. PC lipids were measured by using precursor ion scanning of *m/z* 184; PE lipids (including plasmalogens) were measured by using neutral loss scanning of *m/z* 141; and PS lipids were measured by using neutral loss scanning of *m/z* 185. All species detected for each group are represented as a relative percentage of the sum based on their response values. Abundances of lipid molecular species were calculated by using the Lipid Mass Spectrum Analysis (LIMSA) software (University of Helsinki, Helsinki, Finland). LIMSA was developed at the University of Helsinki for quantitative analysis of mass spectra of complex lipid samples. LIMSA can do peak finding, integration, assigning, isotope correction, and quantitation with internal standards. In this work, raw data from the instrument were exported into Excel, and LIMSA was used as an isotope correction algorithm. Specifically, the method used the integrated area of the first isotope peak and corrected for the isotope overlap by scaling and subtracting the calculated isotope pattern from subsequent peaks. LIMSA then calculated the isotope-corrected abundances by comparison to added internal standards [1.0 µM PC (14:0/14:0), 1.0 µM PE (14:0/14:0), 0.33 µM PS (14:0/14:0), and 12.5 nM ceramide (d18:1/12:0)].

### 2D-TLC and FAME determination of total PC, PE, and PS

Total lipids from tissues were extracted in chloroform-methanol-water (1:1:1) according to the method of Bligh and Dyer (52) as described in Martin et. al. (53) The total lipid extracts were concentrated and stored at −20°C under N_2_ in a known volume of chloroform-methanol (2:1, v/v).

PC, PE, and PS lipid classes were isolated from the total lipid extracts by using high-performance TLC (HPTLC) plates (Analtech, Newark, DE) and a 2D, three-solvent method described previously (53–56). Lipid spots on the HPTLC plates were visualized under UV after staining with 2,7-dichlorofluorescein. The PC, PE, and PS spots were scraped from the plate for gas chromatographic analysis of FAs.

Dichlorofluorescein-stained lipid spots were scraped from the TLC plates, and esterified FAs were hydrolyzed and converted to methyl esters for GC. Silica from each spot was added to a screw-top test tube, and a mixture of pentadecanoic acid (15:0) and heptadecanoic acid (17:0) was added as internal standards. FA methyl esters (FAMEs) were formed by heating in the presence of 2% sulfuric acid in methanol at 85°C for 1 h. FAMEs were extracted into hexane, dried under nitrogen, resuspended in nonane, and quantified by using an Agilent Technologies 7890 gas chromatograph (Agilent Technologies, Santa Clara, CA) with flame ionization detector (57).

### Statistical analysis

Each value presented in the figures is the mean ± SD of four independent analyses. Two-way ANOVA with Tukey’s multiple comparisons test was performed on all molecular species comparisons. Total lipid-phosphorus values reported in supplemental Fig. S1 and supplemental Table S23 are mean values for each region at all ages (*n* = 12). After failing to pass a Brown-Forsythe test of equal SD between each region, raw data for this comparison were transformed to log_10_ [*Y* = Log(*Y*)], and subsequent *P* values and significance were derived from these transformed values. All analysis was performed by using GraphPad Prism (Version 6.07 for Windows, GraphPad Software, San Diego, CA). Error bars for all figures represent SD of the mean; multiplicity-adjusted *P* values are reported in the supplemental tables for each comparison. The tables do not contain the calculated SD values, but these can be derived from the raw data, which will be made available upon request to R.E.A.

## RESULTS

**Fig. 1.**
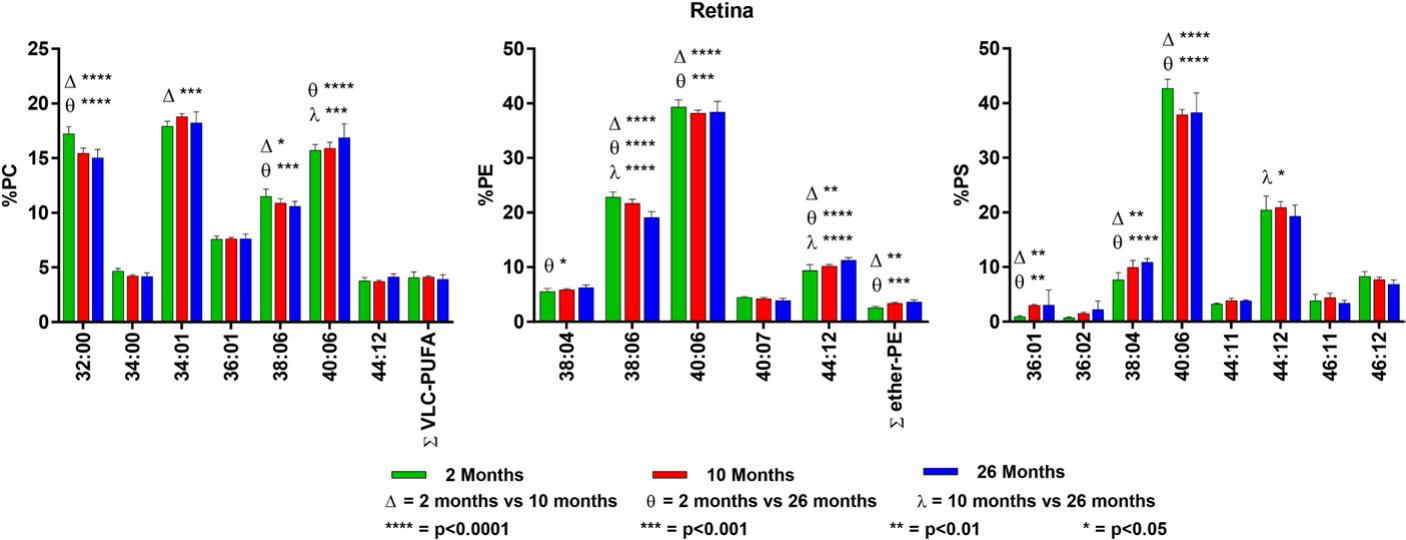
Changes in GPL molecular species composition in retina with age. Looking at major molecular species (≥ 4% abundance) contained within GPLs: PC (left), PE (middle), and PS (right). Retinal tissue was taken at 2, 10, and 26 months of age. Statistics were calculated by using two-way ANOVA with Tukey’s multiple comparisons test. Full list of age-related changes for all molecular species detected can be found in supplemental Tables S1–S3.

**Fig. 2.**
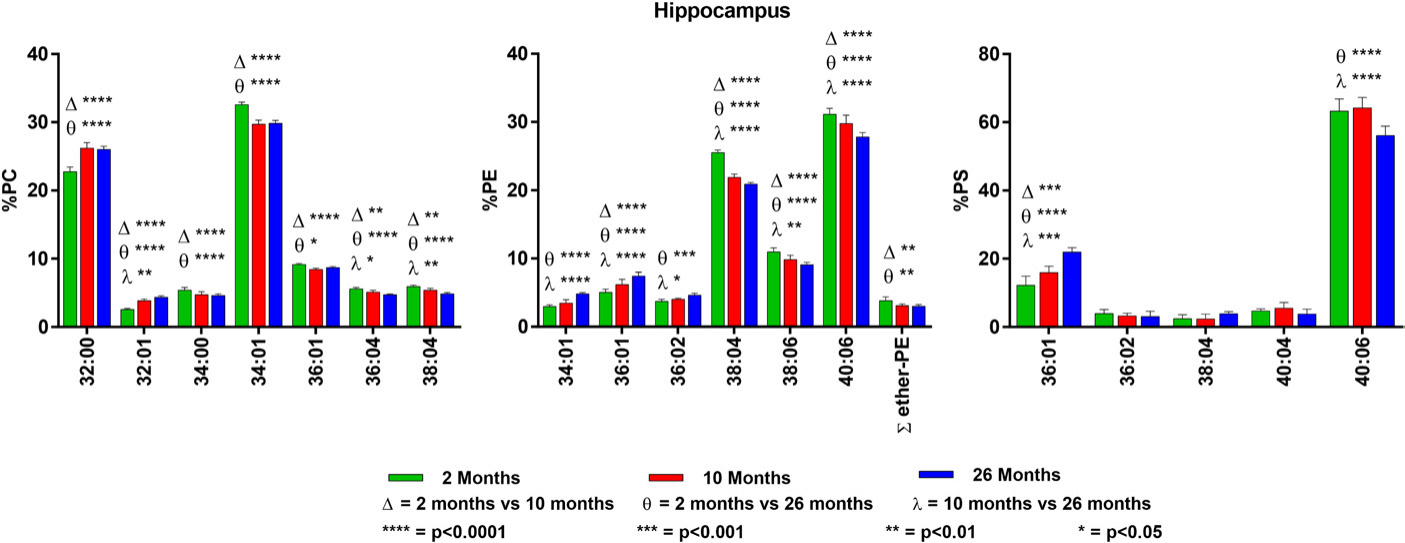
Changes in GPL molecular species composition in hippocampus with age. Looking at major molecular species (≥ 4% abundance) contained within GPLs: PC (left), PE (middle), and PS (right). Hippocampal tissue was taken at 2, 10, and 26 months of age. Statistics were calculated by using two-way ANOVA with Tukey’s multiple comparisons test. Full list of age-related changes for all molecular species detected can be found in supplemental Tables S4–S6.

**Fig. 3.**
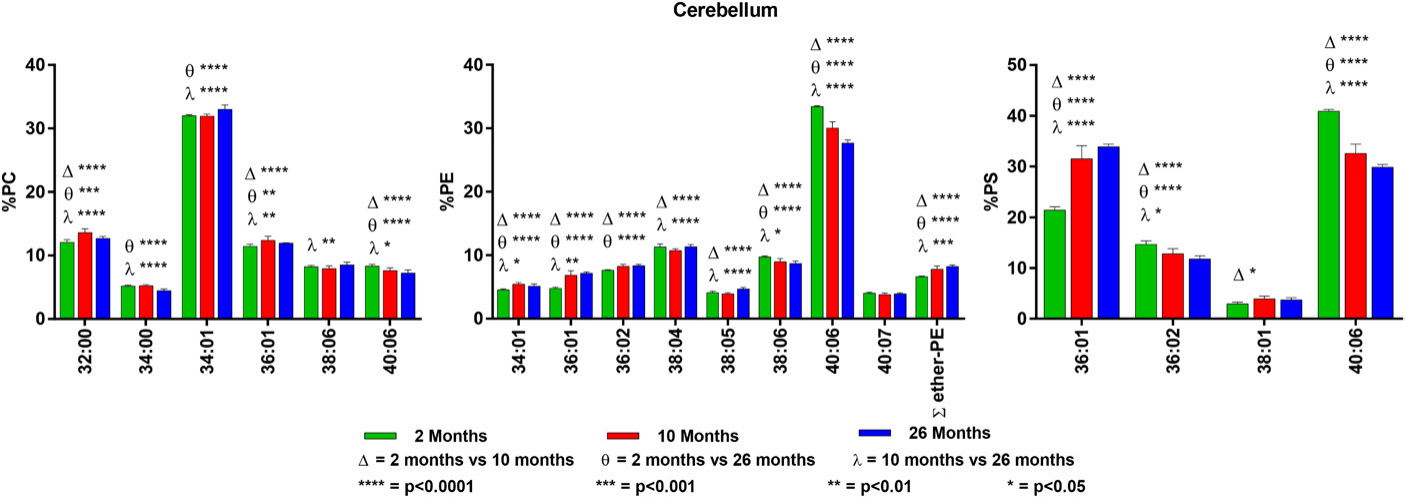
Changes in GPL molecular species composition in cerebellum with age. Looking at major molecular species (≥ 4% abundance) contained within GPLs: PC (left), PE (middle), and PS (right). Cerebellar tissue was taken at 2, 10, and 26 months of age. Statistics were calculated by using two-way ANOVA with Tukey’s multiple comparisons test. Full list of age-related changes for all molecular species detected can be found in supplemental Tables S7–S9.

**Fig. 4.**
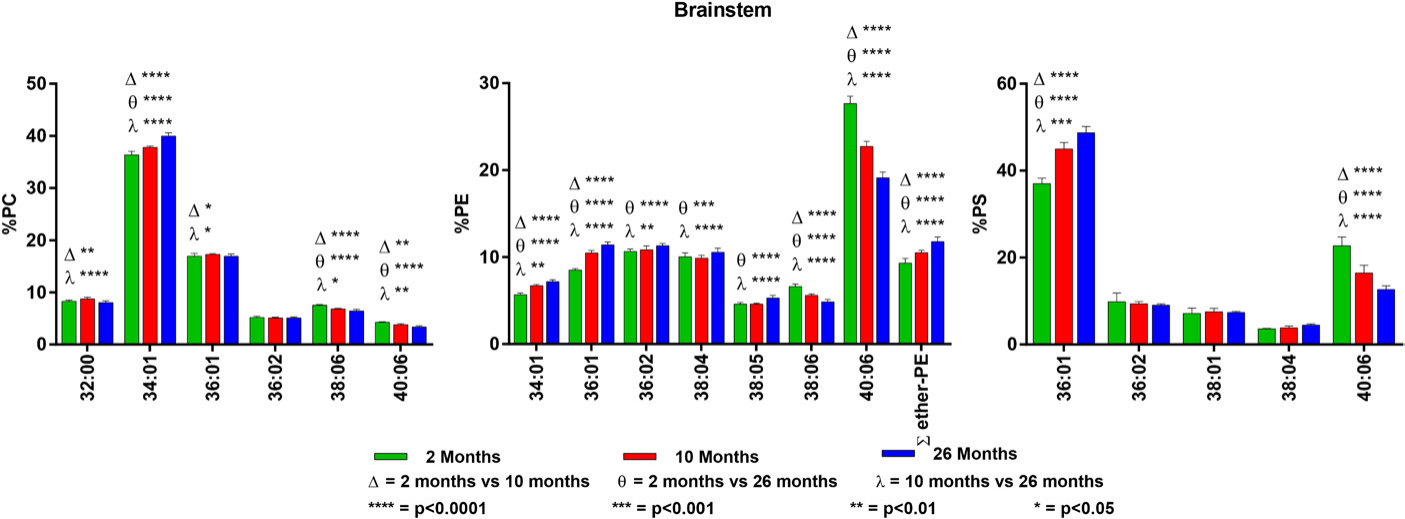
Changes in GPL molecular species composition in brainstem with age. Looking at major molecular species (≥ 4% abundance) contained within GPLs: PC (left), PE (middle), and PS (right). Brainstem tissue was taken at 2, 10, and 26 months of age. Statistics were calculated by using two-way ANOVA with Tukey’s multiple comparisons test. Full list of age-related changes for all molecular species detected can be found in supplemental Tables S10–S12.

**Fig. 5.**
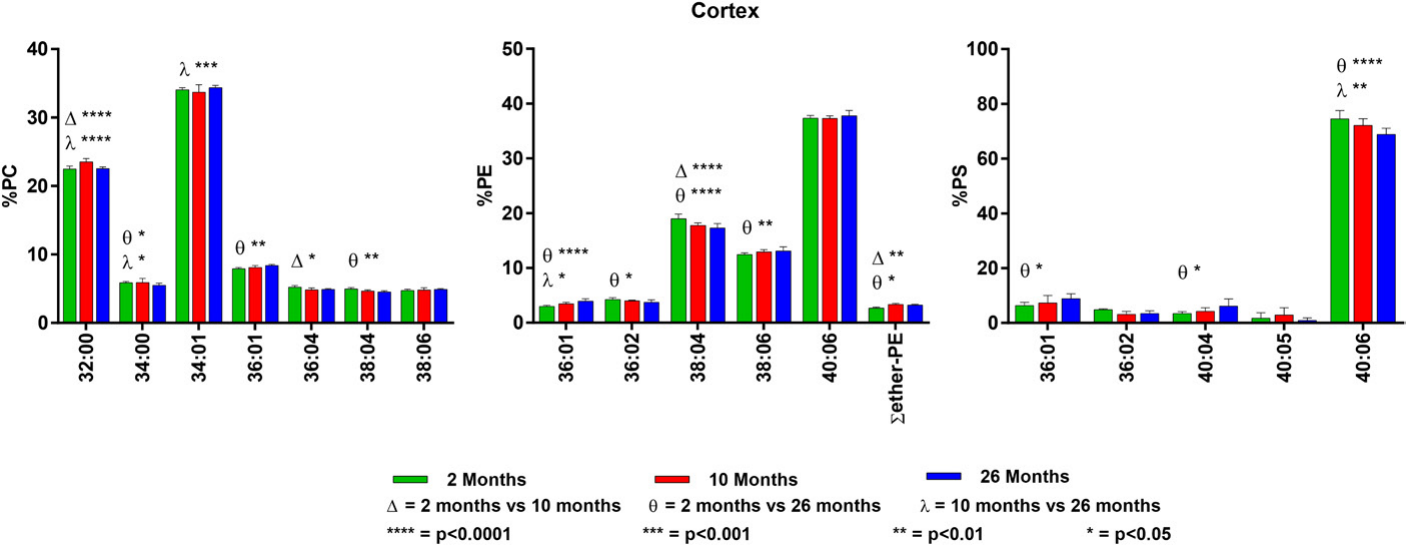
Changes in GPL molecular species composition in cortex with age. Looking at major molecular species (≥ 4% abundance) contained within GPLs: PC (left), PE (middle), and PS (right). Cortical tissue was taken at 2, 10, and 26 months of age. Statistics were calculated by using two-way ANOVA with Tukey’s multiple comparisons test. Full list of age-related changes for all molecular species detected can be found in supplemental Tables S13–S15.

**Fig. 6.**
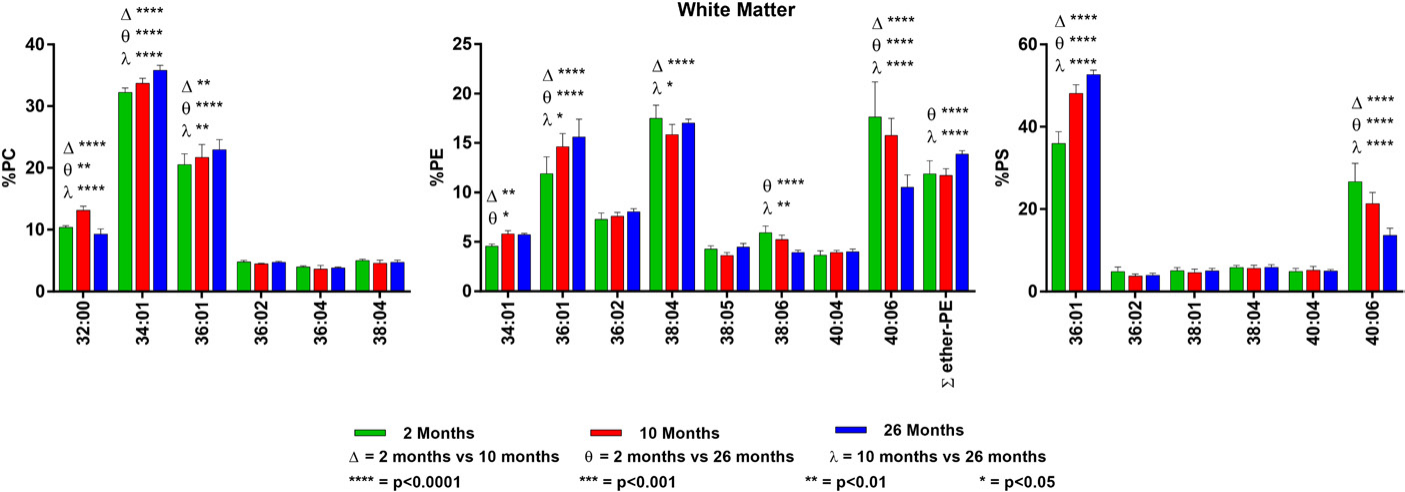
Changes in GPL molecular species composition in white matter with age. Looking at major molecular species (≥ 4% abundance) contained within GPLs: PC (left), PE (middle), and PS (right). White matter tissue was taken at 2, 10, and 26 months of age. Statistics were calculated by using two-way ANOVA with Tukey’s multiple comparisons test. Full list of age-related changes for all molecular species detected can be found in supplemental Tables S16–S18.

**Fig. 7.**
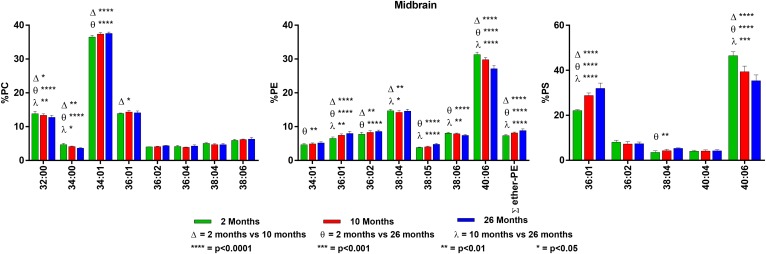
Changes in GPL molecular species composition in midbrain with age. Looking at major molecular species (≥ 4% abundance) contained within GPLs: PC (left), PE (middle), and PS (right). Midbrain tissue was taken at 2, 10, and 26 months of age. Statistics were calculated by using two-way ANOVA with Tukey’s multiple comparisons test. Full list of age-related changes for all molecular species detected can be found in supplemental Tables S19–S21.

Analysis of PC, PE, and PS was performed in retina, hippocampus, cerebellum, brainstem, cortex, white matter, and midbrain of 2-, 10-, and 26-month-old mice. The relative percentages of each molecular species of PC, PE, and PS were compared for each age, and those present at 4% abundance or greater were graphed along with PE plasmalogens for each tissue (**Figs. 1–7**). The relative percentage of all molecular species in each GPL class and the statistical analysis of changes with age are presented in supplemental Tables S1–S21. In addition, we measured total nanomoles lipid-phosphorus per milligram of wet tissue weight for each region at each age (supplemental Table S22). After determining no age-related differences within the same tissue (except for a *P* = 0.048 for 2 vs. 26 months in white matter), we collapsed the age groups and compared each region. This revealed significant differences in total lipid-phosphorus between some of the regions (supplemental Fig. S1 and supplemental Table S23). PC, PE, and PS were quantified by 2D-TLC and are presented both as nanomoles per milligram of wet weight and as the relative mol% of each to the others (supplemental Tables S24–S26). There was a significant age-related loss of PC and a relative increase in PE in both white matter and cortex. There were no age-related changes in PS for any tissue.

Examination of the specific GPL classes revealed that each had a unique molecular composition that, in many cases, changed significantly with age. PC contained the largest percentage of shorter-chain saturated (SAT) and monoenoic (MONO) molecular species, whereas PE and PS were predominantly composed of species containing PUFAs. Only the retina contained a high percentage of di-PUFA species, primarily in PS. There were significant age-related changes in the molecular species composition across most regions, with the relative levels of SAT and MONO species increasing with age at the expense of those containing PUFA. There were also regional differences within the same GLC class, with the tissues most abundant in neurons and synapses (i.e., retina and hippocampus) containing the highest levels of PUFA species, whereas the tissues most abundant in myelin (i.e., white matter and brainstem) contained the highest levels of SAT and MONO species. These tissues high in myelinated fibers also contained the highest levels of total lipid-phosphorus per milligram of wet weight.

### Retina

PC has a large amount of di-SAT (32:00, 16:0/16:0) and SAT/MONO (34:01, 16:0/18:1) molecular species, which are relatively low in PE and PS. Of all the tissues and classes analyzed, retinal PC is the only class to contain detectable levels of very-long-chain PUFA (VLC-PUFA; 28 carbons in length and typically containing both n3 and n6 PUFA). VLC-PUFA makes up approximately 4% of the total PC isolated from retina, and these levels seem to remain stable in the retina with age (Fig. 1 and supplemental Table S1). The major molecular species in PE and PS is 40:06 (18:0/22:6n3). Retina is unique among tissues in that PC, PS, and PE contain di-PUFA species, with PS having the largest amount (∼30%). This is in stark contrast with brain tissues, which contain much smaller amounts (ranging approximately from 0.6–2.9%). PE is the only lipid class to contain significant amounts of vinyl ethers (plasmalogens), which are much lower in the retina than in most brain tissues (approximately 2.6% compared with 12% in white matter).

There were minimal significant age-related changes in the GPL molecular species composition of retina. There was a modest decrease in 32:00 and 38:06 and a concomitant increase in 40:06 in PC (Fig. 1 and supplemental Table S1). In PE, there was a small age-related decrease in 40:06 and compensatory increase in 44:12 (22:6n3/22:6n3). Notably, there was an age-related increase in the levels of PE plasmalogens with age (Fig. 1 and supplemental Table S2). PS shows a significant age-related increase in the shorter-chain FA-containing molecular species [34:01 and 38:04 (18:0/20:4n6)] and a subsequent decrease in 40:06 and 44:12 (Fig. 1 and supplemental Table S3).

The age-related changes in molecular species composition in retinal GPLs are small compared with the changes we found in all regions of the brain.

### Hippocampus

PC is composed primarily of di-SAT and SAT/MONO species, with only approximately 18% of the species containing PUFA, with ∼12% of 20:4n6- and ∼6% of 22:6n3-containing species. In contrast, both PE and PS contain high levels of 40:06 (18:0/22:6n3), which make up ∼60% of PS and ∼30% of PE. Plasmalogens make up approximately 2.5% of hippocampal PE, which is among the lowest levels found in brain and similar to retina. PS contains only small amounts of 20:4n6-containing species (approximately 7%), whereas PE species contain almost 30% of 20:4n6.

There were small, but significant, age-related changes in several of the hippocampal GPL species, which, in general, were an increase in the SAT-containing species and reduction in the PUFA-containing species. In PC, there was a slight increase in 32:00 and 32:01 at the expense of 34:01, 36:04, and 38:04. There were no age-related changes in PC species containing 22:6n3 (Fig. 2 and supplemental Table S4). PE also had an age-related increase in the SAT- and MONO-containing molecular species (34:01 and 36:01), with a concomitant decrease in the PUFA-containing molecular species (38:04, 38:06, and 40:06). Of interest, hippocampus was the only tissue to show an age-related decline rather than an increase in the percent plasmalogens in PE (Fig. 2 and supplemental Table S5).

PS showed the greatest age-related increase in the shorter-chain SAT/MONO FA-containing species 36:01 (12% at 2 months vs. 22% at 26 months), with a parallel decrease in the 22:6n3 species 40:06 (63% at 2 months vs. 56% at 26 months) (Fig. 2 and supplemental Table S6).

### Cerebellum

PC was primarily composed of di-SAT and SAT/MONO species, with the most predominant at 2 months of age being 34:01. Species containing 22:6n3 were ∼19% of the total and those containing 20:4n6 were ∼8%. The major molecular species in PE and PS was 40:06. However, PS also contained high levels of 36:01 (18:0/18:1). The combination of 36:01 and 40:06 was also found in PS from brainstem, white matter, and midbrain. Plasmalogens make up approximately 7% of cerebellar PE.

There were significant age-related changes in many of the GPL molecular species in the cerebellum. Although statistically significant (supplemental Table S7), the changes in PC were small and reflected a slight increase in the SAT and SAT/MONO species at the expense of those containing PUFA. More dramatic changes were found in PE and PS. In PE, there was a decrease in the major 22:6n3-containing species and an increase in the SAT and SAT/MONO species. There was also an age-related increase in PE plasmalogens. PS had the greatest age-related changes, with an increase in 36:01 from 21 to 34% (2 vs. 26 months) and a decrease in 40:06 from 41 to 30%. For details on the cerebellum findings, see Fig. 3 and supplemental Tables S8 and S9.

### Brainstem

Major GPL molecular species in the brainstem closely resemble those found in the cerebellum with the exception of an interesting shift in PS from 40:06 as the dominant species to 36:01 as the most abundant. Plasmalogens make up approximately 9% of brainstem PE. There were significant age-related changes in all three GPL classes, with changes in PE and PS being greater than those in PC. All glycerolipid classes again showed an age-related increase in shorter chain di-SAT- and SAT/MONO-containing molecular species, with an associated reduction in the PUFA-containing molecular species. PC showed the same type of age-related changes noted previously in the cerebellum, with the most notable being a significant increase in 34:01 and a decrease in 40:06. PE had significant age-related changes in all major molecular species, with increases in 34:01 and 36:01 and a large decrease in 40:06 (28% at 2 months to 19% at 26 months). Similar to the other tissues except the hippocampus, there was a significant age-related increase in brainstem plasmalogens. For details on the brainstem findings, see supplemental Tables S10 and S11.

The molecular species in brainstem PS were quite different from PS in any of the other tissues in that the major species is the SAT/MONO 36:01, which was 37% at 2 months and increased to 49% at 26 months. The species 40:06, the predominant species in the other regions, was 23% at 2 months and decreased to 13% at 26 months (Fig. 4 and supplemental Table S12).

### Cortex

The majority of PC is made up of di-SAT (29%) and SAT/MONO (40%) molecular species, with only ∼8% containing 22:6n3. As found in other tissues, the largest molecular species in PE is 40:06, followed by 38:04. Unlike most other tissues, the level of PE plasmalogens was quite low (3%). The most surprising finding was the very large amount of 40:06 in PS (75% at 2 months), which was greater than that found in hippocampus (63%). Although there were significant age-related changes that favored an increase in SAT and SAT/MONO species at the expense of PUFA species, the magnitude of these changes was similar to the small changes noted in the retina and hippocampus, compared with the larger changes found in the other brain regions. For details on the cortex findings, see Fig. 5 and supplemental Tables S13–S15.

### White matter

In general, the GPL in white matter contained more SAT-containing molecular species compared with most of the other regions. In addition, although the overall amounts of PUFA were low, it was interesting to note that, in white matter, the major PUFA present was 20:4n6 instead of the more typical 22:6n3. PC composition of white matter was similar to that seen in the brainstem and cerebellum, with high levels of 32:00, 34:01, and 36:01 (combined 63% at 2 months) and low levels of PUFA species containing 22:6n3 (∼5%). PE contained equal amounts (18%) of 38:04 (18:0/20:4n6) and 40:6 (18:0/22:6n3), which was not the case in the other regions. White matter contained the highest percent of PE plasmalogens of all brain regions analyzed (12%). PS in white matter more closely resembled that of the brainstem, with 36:01 as the most abundant molecular species over 40:06 (36 and 27%, respectively).

There were small, but significant, age-related changes in PC. The di-SAT species 32:00 and 34:00 were reduced with age, and the SAT/MONO species 34:01 and 36:01 were increased. The changes in PE were of greater magnitude, with 36:01 increasing from 12 to 16% and 40:06 decreasing from 18 to 11%. The PE plasmalogens also increased with age. The largest changes were in PS, where 36:01 increased from 36 to 53% with age, whereas 40:06 decreased from 27 to 14%. For details on the white matter findings, see Fig. 6 and supplemental Tables S16–S18.

### Midbrain

Midbrain contains a lipid composition similar to that of the cerebellum and brainstem. PC is made up of di-SAT (32:00) and SAT/MONO (34:01 and 36:01) molecular species and low levels of species containing 22:6n3 (12%). The PE molecular species in 2-month-old midbrain were 38:04 (15%) and 40:06 (31%). There were small age-related changes, with increases in the SAT/MONO species and decreases in the 22:6n3-containing species. Plasmalogens make up ∼7% of midbrain PE and increased slightly, but significantly, with age. We found that 40:06 was the most abundant molecular species in midbrain PS (47% at 2 months of age), with 36:01 present at 22%.

There were statistically significant, but minor, age-related changes in the PC molecular species (±1%), with the more saturated species increasing with age. PE showed similar minor changes with age. However, PS, as found in most of the other regions, underwent dramatic age-related changes, with 36:01 increasing from 22 to 32% and 40:06 decreasing from 47 to 35%. For details on the midbrain findings, see Fig. 7 and supplemental Tables S19–S22.

## DISCUSSION

The goals of this study in testing our initial hypothesis were to provide a novel and all-inclusive molecular blueprint of the age-related changes in the composition of the three major GPL classes in the retina and specific brain regions. The significance of the work done here is that it provides a high level of detail regarding every detectable molecular species of the three major brain and retina GPLs in a single source. The supplemental tables provide detailed compositional information on the retina and brain lipidome in its detectable entirety. Our study confirms that the various regions of the brain contain unique compositions of lipid molecules and that this composition changes in a molecule- and region-specific manner with age.

The most prevalent and consistent findings were as follows: *1*) age-dependent increases in SAT-containing molecular species at the expense of those containing PUFA, especially DHA; *2*) dramatic differences in the molecular species composition of the three GPL classes in each region; and *3*) large regional differences in the molecular species composition of each GPL class, with gray matter-dominant tissues having higher levels of PUFA-containing molecular species.

### PUFA-containing GPLs are reduced in myelin-rich regions, whereas SATs are elevated, suggesting the need for unique molecular compositions depending on regional function

**Fig. 8.**
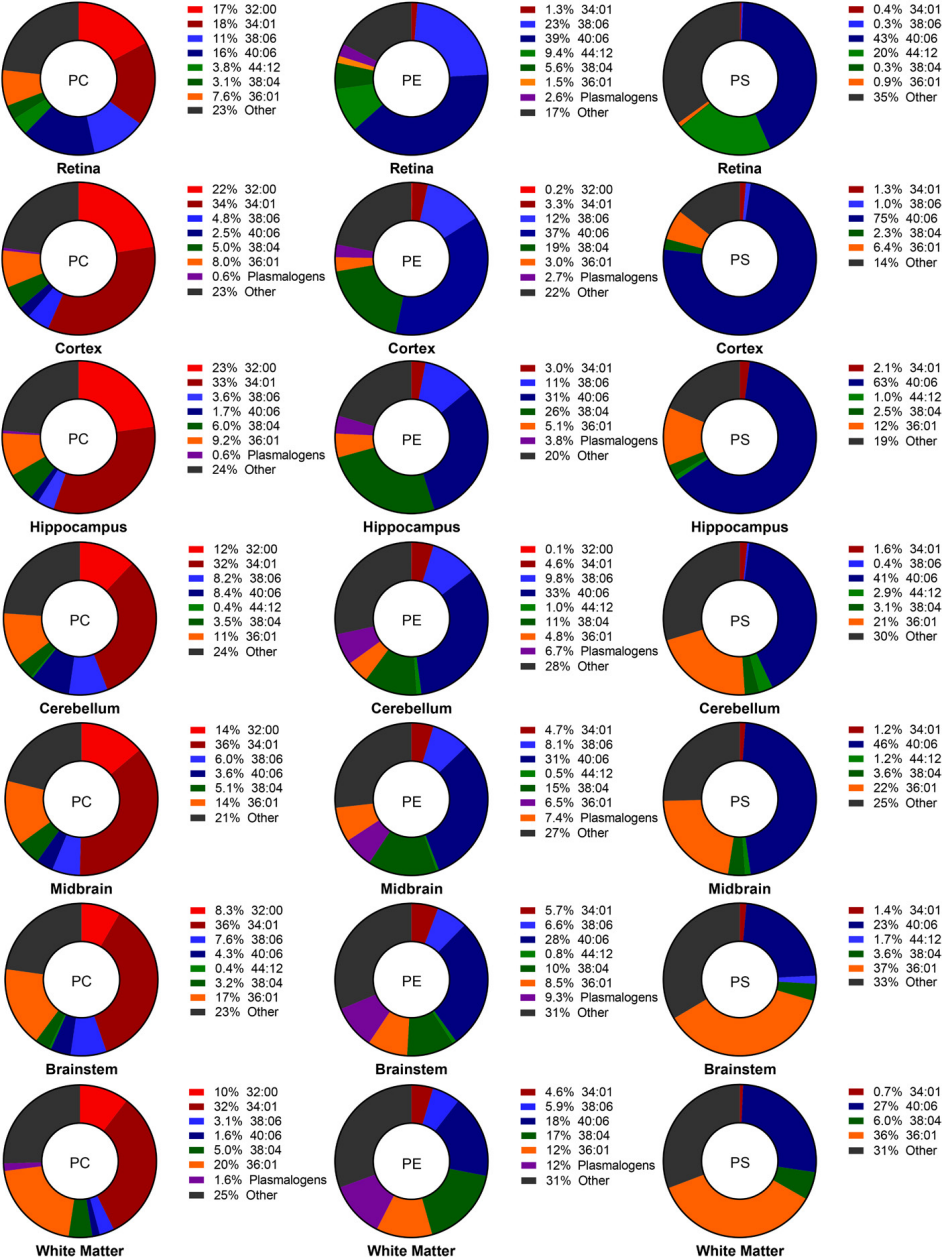
Percent composition of major molecular species across region and glycerolipid class at 2 months of age. Ordered from top to bottom with increasing white matter content. Values of the major molecular species chosen are expressed as percentages with other molecular species detected summed as “other.” Significant age-related changes in all molecular species for all three classes of glycerolipids can be found in supplemental Tables S1–S21.

The molecular species for each GPL class in the different regions of 2-month-old brain and retina are shown in **Fig. 8**, arranged in descending order from lowest to highest levels of myelin. Within each GPL class, there was a pronounced change in the relative molecular species composition between the different tissues, with the levels of PUFA-containing species dramatically lower in the tissues containing the highest levels of myelin. This is most evident when cortex and white matter are compared, where the sum of 38:06 and 40:06 ranged from 49% (PE) and 76% (PS) in cortex to 24% and 33%, respectively, in white matter. Alterations in the lipid composition of the same class by region may be indicative of the membrane fluidity needed by that region to function properly. The high incorporation of DHA and the other PUFAs in regions like the retina, cortex, hippocampus, and cerebellum may result in more “plastic” membranes, allowing for improved information processing and synaptic function (58–64). Conversely, the increased levels of SAT-containing species in the myelin-rich tissues supports their role in providing insulation for the nerve fibers. Understanding the possible influence of these specific molecules on neural membranes and the known functional outputs of the regions in which they are located will lead to a better understanding of the dramatic compositional and functional differences we observe in the brain as a whole. Because these molecular species change with age, any influence they may have on cellular function may also change.

### DHA is an essential, life-giving FA that we cannot make

The major PUFAs in the brain and retina are DHA (22:6n3) and AA (20:4n6), both of which are essential FAs because they cannot be synthesized by any vertebrate, but by only the lower forms of invertebrates (e.g., *Caenorhabditis* *elegans*). Mammals obtain DHA and AA from their diet or from hepatic conversion of shorter-chain PUFAs, such as linoleic (18:2n6) and linolenic (18:3n3) acids (60, 65). Although the levels of DHA and, to some extent, AA are relatively low in the blood, the retina and brain are able to take them up and incorporate them into the various molecular species reported in the supplemental tables. This enrichment of DHA and AA in retina and brain lipids begins in utero and is essential for normal brain and retina development and function. Once incorporated into these organs, DHA and AA are tenaciously retained and cannot be depleted by removing all dietary sources of n3 and n6 PUFA. Thus, any age-related changes in DHA- and AA-containing molecular species are due to specific events in brain and retina, and are not due to dietary restrictions, which should not have a demonstrable effect on the brain and retina FA compositions measured in this study.

A study of more than 6,000 individuals over the age of 65 indicated that increasing DHA levels with a high fish diet had a protective effect on cognitive decline (66, 67). Work by Bazan (13, 14) demonstrated that this is likely, in part, due to DHA serving as a precursor for bioactive lipid derivatives like neuroprotectant-D1, which has been shown to have an extensive and beneficial role in both brain and retina neurons and may protect against age-related cognitive decline and other neurodegenerative diseases.

Loss of the molecular species that contain DHA with age was the most consistent finding across brain regions. DHA has been studied extensively in brain health and function as a neuroprotectant and has been linked to many important neural processes that span from early development to death (68). The highest concentrations of DHA molecular species exist in PE and PS and are enriched in synaptic membranes, followed by mitochondrial membranes, and finally microsomal membranes (60, 69). DHA enriched membranes are thin due to DHA’s 3D conformation via its six methylene-interrupted *cis* double bonds (70). The fact that DHA undergoes rapid interconversion between various states of torsion results in membranes with increased permeability, compression, fusion, and flipping properties (62, 63, 71). The presence of DHA has also been shown to drive the generation of cholesterol-depleted domains (64), and, by favoring insertion into cholesterol-rich lipid raft domains, DHA promotes activities such as neurotransmitter release, second messenger signaling, resistance to oxidative stress, and even gene regulation (58, 61). In other words, loss of DHA-containing molecular species with age results in more rigid, less fluid neural membranes. We would predict that noted age-related declines in cognitive function could be due, at least in part, to changes in the synaptic apparatus due to reduced DHA molecular species. Increased brain accumulation of DHA from diet in rodents resulted in higher levels of both presynaptic and postsynaptic proteins critical for neurotransmission, including syntaxin-3, PSD-95, and synapsin-1 (72). Indeed, VanGuilder et al (73) demonstrated that synaptosomes isolated from young, adult, and aged Wistar rats had significant decreases with age in SNAP25, synaptophysin, synaptic vesicle glycoprotein 2B, SV2-related protein, Homer 1, and synaptoporin, all of which are critical for neurotransmission. Finally, electron microscopy of synaptic vesicle membranes isolated from these animals appeared to lose their structural and morphological integrity with age (74). These findings support the concept that initial changes in the lipidome could be driving the loss of membrane stability and integrity that subsequently deregulates the protein machinery necessary for synaptic transmission.

Another hypothesis for DHA’s high incorporation into neural membranes was proposed by Crawford et al. (59) in a paper describing a quantum theory for DHA’s role in the brain, in that its unique molecular structure allows for quantum transfer of its π-electrons between neural membranes as a form of intercellular communication. DHA is also involved in the regulation and biosynthesis of PS in that high levels of DHA correlate with increased biosynthesis of PS (75), which has been linked to a positive effect on neuronal function and survival via the PI 3-kinase/Akt pathway (76).

Given these multifaceted and important roles for DHA (77), it is problematic that we are reporting a consistent age-related loss of the 40:06 molcular species from PC in both the cerebellum (*P* < 0.0001) and brainstem (*P* < 0.0001). In addition, significant age-related reduction of 40:06 was observed in PE in retina and in every brain region except cortex, and significant reduction of 40:06 from PS was observed in every brain region, as well as in retina. We found that 38:06 (16:0 + 22:6n3), another relevant DHA-containing molecular species, was significantly reduced with age in PC in retina, brainstem, and white matter, and in PE in retina and every brain region except cortex. These age-related changes could have profound effects on synaptic function and cognitive ability.

### Although immensely important in neural function for the retina, VLC-PUFAs were not detected in the rodent brain, except for trace amounts during embryogenesis

Another important class of FAs are the VLC-PUFAs (28 carbons) and very-long-chain saturated FAs (primarily 28:0 and 30:0). These FAs are synthesized exclusively by ELOVL4, a condensing enzyme that catalyzes the rate-limiting first step in their biosynthesis from C-26 precursors (78). ELOVL4 is expressed in retina (78–82) and brain (83–87), as well as in other tissues including skin (88–91), testes (92), and Meibomian glands (93). In the retina, the major product is VLC-PUFA, which is found exclusively in the *sn-1* position of PC (94). Because the brain expresses ELOVL4 and has such high levels of PUFA, we anticipated finding VLC-PUFA in brain PC. However, we were surprised to find no VLC-PUFA in PC in any brain region at any of the three ages we examined. Because Poulos and coworkers (32) had reported finding VLC-PUFA in neonatal rat brain, we dissected whole brain from postnatal day 1 (P1) rats, isolated PC by TLC, and examined methyl esters by GC/MS, as Poulos and coworkers had done. We were unable to detect even the smallest amounts of any VLC- PUFA, despite easy detection of VLC-PUFA in PC from retina as a positive control. We also examined the upper phase of the Folch extract, as well as the protein interface, for any potentially protein-bound VLC-PUFA, but again we found none. The highest embryonic expression of *Elovl4* in mouse brain (95) is during late embryonic development, with a rapid loss of *Elovl4* mRNA from P1–P30. We dissected hippocampus from embryonic day 18.15 (E18.5) rat brains as well as hippocampus, cortex, cerebellum, and whole brain from the E18.5 embryonic mouse. In the total lipid extracts of each of these regions, we did not find any detectable levels of VLC-PUFA, except for possibly trace amounts of a single VLC-PUFA peak. It is possible that, early in embryonic development, these molecules exist in the brain at very low abundance beyond the bounds of quantitative detection. If so, they could have an important, but transient, role in brain development, perhaps acting as precursors for other bioactive derivatives, as has been reported for DHA (13, 96, 97). Interestingly, we found significant amounts of 28:0 and 30:0, both products of ELOVL4, in sphingolipids in all regions of the brain and in the retina. These findings will be discussed in a subsequent paper.

### Plasmalogens are neuroprotective molecules that influence numerous dynamic cellular functions, and their loss results in both retinal and brain pathologies of a severe nature

PE is the only lipid class in which we detected significant amounts of plasmalogens. Plasmalogens are unique PE lipid molecules that contain an ether-linked alk-1-enyl chain with a *cis* double bond, termed a “vinyl-ether linkage,” at the *sn*-1 position instead of the typical ester-linked FAs found in other GPL molecules (98–100). It is the presence of this vinyl ether double bond that makes these plasmalogens so uniquely sensitive to acid, mercury cations, and reactive oxygen species (15, 101–108) and, as a result, important for the aging organism. Patients with an inability to synthesize plasmalogens are left with a wide variety of pathologies, including severe mental retardation, hypotonicity, adrenal dysfunction, cataracts, deafness, facial dysmorphism, chondrodysplasia, and very early mortality, often within the first year of life (109). The brain region with the highest percent of ether-PE plasmalogens is white matter (<12%), with cortex being the lowest (<3%); retina was the lowest of all tissues measured (2.5%).

Plasmalogens have been shown to play a unique role in maintaining the biophysical properties of the membranes in which they are expressed. Their presence appears to facilitate membrane fluidity, and they have been suggested to play a role in membrane fusion and perhaps in mediating vesicle fusion (110). Plasmalogens have also been reported to increase or decrease certain protein kinase C-mediated responses in various models, which are well-known contributors to learning and memory circuitry in the hippocampus (111–116). Thus, plasmalogens are important for neurotransmission (117–119). The hippocampus is uniquely sensitive to an age-related decline in function, with reports of short-term memory loss as a consequence of normal aging (120–124). These hippocampal-mediated effects are even more profound in patients with various forms of cognitive impairment, and the hippocampal pathologies of Alzheimer’s disease have been a significant focus in the field for the last several decades. Transient ischemia is another risk factor for older individuals and is a cause of vascular-related dementias over time due to continuous oxidative stress. Plasmalogens have been shown to have a protective role in response to cellular oxidative stress during ischemia-reperfusion injury (103, 125). The hippocampus was the only brain region to show a significant, albeit small, age-related reduction in PE plasmalogens, whereas every other tissue demonstrated the opposite response. Given the well-documented neuroprotective roles of both DHA and plasmalogens, and the significant loss of both from the hippocampus alone, their reduction could be important in the context of age-related changes in cognition.

### Lipids are dynamic and influential molecules that deserve our attention as neuroscientists

Piomelli et al. (126) stated in 2007 that, “Neuroscientists have a problem with fat.” As a whole, the field of neuroscience has neglected lipids, presuming that these critical components of the cell were not dynamic, but meant solely for membrane structure and axon insulation. With the incredible advances in our understanding of both the nervous system as a whole and having the tools for precise measurement of lipid species, we are now able to address questions whose answers were heretofore not attainable. The molecular blueprint we present here, we hope, will provide a template for scientists to ask specific questions targeting individual lipid molecules and uncover their region-specific or ubiquitous functions in the nervous system.

## Supplementary Material

Supplemental Data

## References

[1] Yehuda S., Rabinovitz S., Carasso R. L., and Mostofsky D. I. 1998 Fatty acids and brain peptides. Peptides. 19: 407–419. 9493877 10.1016/s0196-9781(97)00295-7

[2] Langelier B., Linard A., Bordat C., Lavialle M., and Heberden C. 2010 Long chain-polyunsaturated fatty acids modulate membrane phospholipid composition and protein localization in lipid rafts of neural stem cell cultures. J. Cell. Biochem. 110: 1356–1364. 20564231 10.1002/jcb.22652

[3] Chemin J., Cazade M., and Lory P. 2014 Modulation of T-type calcium channels by bioactive lipids. Pflugers Arch. 466: 689–700. 24531745 10.1007/s00424-014-1467-5

[4] Carta M., Lanore F., Rebola N., Szabo Z., Da Silva S. V., Lourenco J., Verraes A., Nadler A., Schultz C., Blanchet C., 2014 Membrane lipids tune synaptic transmission by direct modulation of presynaptic potassium channels. Neuron. 81: 787–799. 24486086 10.1016/j.neuron.2013.12.028

[5] Moreno C., Macias A., Prieto A., De La Cruz A., and Valenzuela C. 2012 Polyunsaturated fatty acids modify the gating of kv channels. Front. Pharmacol. 3: 163, 1–8. 10.3389/fphar.2012.00163 PMC3437463 22973228

[6] Vreugdenhil M., Bruehl C., Voskuyl R. A., Kang J. X., Leaf A., and Wadman W. J. 1996 Polyunsaturated fatty acids modulate sodium and calcium currents in CA1 neurons. Proc. Natl. Acad. Sci. USA. 93: 12559–12563. 8901621 10.1073/pnas.93.22.12559 PMC38031

[7] Yeh G. C., Wang S. M., and Wang I. F. 1995 Interaction of arachidonic acid with ligand binding sites of the N-methyl-D-aspartate receptor in rat hippocampal membranes. Chin. J. Physiol. 38: 117–123. 8697896

[8] Kays J. S., Li C., and Nicol G. D. 2012 Expression of sphingosine 1-phosphate receptors in the rat dorsal root ganglia and defined single isolated sensory neurons. Physiol. Genomics. 44: 889–901. 22805346 10.1152/physiolgenomics.00053.2012 PMC3472456

[9] Kajimoto T., Okada T., Yu H., Goparaju S. K., Jahangeer S., and Nakamura S. 2007 Involvement of sphingosine-1-phosphate in glutamate secretion in hippocampal neurons. Mol. Cell. Biol. 27: 3429–3440. 17325039 10.1128/MCB.01465-06 PMC1899953

[10] Blom T., Bergelin N., Slotte J. P., and Tornquist K. 2006 Sphingosine kinase regulates voltage operated calcium channels in GH4C1 rat pituitary cells. Cell. Signal. 18: 1366–1375. 16321506 10.1016/j.cellsig.2005.10.014

[11] Titievsky A., Titievskaya I., Pasternack M., Kaila K., and Tornquist K. 1998 Sphingosine inhibits voltage-operated calcium channels in GH4C1 cells. J. Biol. Chem. 273: 242–247. 9417071 10.1074/jbc.273.1.242

[12] Oishi K., Zheng B., and Kuo J. F. 1990 Inhibition of Na,K-ATPase and sodium pump by protein kinase C regulators sphingosine, lysophosphatidylcholine, and oleic acid. J. Biol. Chem. 265: 70–75. 2152929

[13] Bazan N. G. 2005 Neuroprotectin D1 (NPD1) a DHA-derived mediator that protects brain and retina against cell injury-induced oxidative stress. Brain Pathol. 15: 159–166. 15912889 10.1111/j.1750-3639.2005.tb00513.x PMC8095981

[14] Mukherjee P. K., Marcheselli V. L., Serhan C. N., and Bazan N. G. 2004 Neuroprotectin D1 a docosahexaenoic acid-derived docosatriene protects human retinal pigment epithelial cells from oxidative stress. Proc. Natl. Acad. Sci. USA. 101: 8491–8496. 15152078 10.1073/pnas.0402531101 PMC420421

[15] Zoeller R. A., Lake A. C., Nagan N., Gaposchkin D. P., Legner M. A., and Lieberthal W. 1999 Plasmalogens as endogenous antioxidants: somatic cell mutants reveal the importance of the vinyl ether. Biochem. J. 338: 769–776. 10051451 PMC1220115

[16] Barceló-Coblijn G., Hogyes E., Kitajka K., Puskas L. G., Zvara A., Hackler L. Jr., Nyakas C., Penke Z., and Farkas T. 2003 Modif­ication by docosahexaenoic acid of age-induced alterations in gene expression and molecular composition of rat brain phospholipids. Proc. Natl. Acad. Sci. USA. 100: 11321–11326. 13679584 10.1073/pnas.1734008100 PMC208755

[17] Kitajka K., Sinclair A. J., Weisinger R. S., Weisinger H. S., Mathai M., Jayasooriya A. P., Halver J. E., and Puskas L. G. 2004 Effects of dietary omega-3 polyunsaturated fatty acids on brain gene expression. Proc. Natl. Acad. Sci. USA. 101: 10931–10936. 15263092 10.1073/pnas.0402342101 PMC503722

[18] Sampath H., and Ntambi J. M. 2004 Polyunsaturated fatty acid regulation of gene expression. Nutr. Rev. 62: 333–339. 15497766 10.1111/j.1753-4887.2004.tb00058.x

[19] Kodas E., Galineau L., Bodard S., Vancassel S., Guilloteau D., Besnard J. C., and Chalon S. 2004 Serotoninergic neurotransmission is affected by n-3 polyunsaturated fatty acids in the rat. J. Neurochem. 89: 695–702. 15086526 10.1111/j.1471-4159.2004.02401.x

[20] Folch-Pi J. 1959 [Recent studies on the chemistry of the brain and their relation with the structure of the myelin sheath]. Expos. Annu. Biochim. Med. 21: 81–95. 13823691

[21] Khan A. A., and Folch-Pi J. 1967 Cholesterol turnover in brain subcellular particles. J. Neurochem. 14: 1099–1105. 6078588 10.1111/j.1471-4159.1967.tb06155.x

[22] Folch-Pi J. 1968 The composition of nervous membranes. Prog. Brain Res. 29: 1–17. 4309620 10.1016/S0079-6123(08)64146-1

[23] Ansell G. B., and Spanner S. 1963 The occurence of a long-chain ether analogue of phosphatidylethanolamine in brain tissue. Biochem. J. 88: 56–64. 14013279 10.1042/bj0880056 PMC1203847

[24] Ansell G. B., and Spanner S. 1963 The alkaline hydrolysis of the ethanolamine plasmalogen of brain tissue. J. Neurochem. 10: 941–945. 14087698 10.1111/j.1471-4159.1963.tb11921.x

[25] Ansell G. B., and Spanner S. 1967 The metabolism of labelled ethanolamine in the brain of the rat in vivo. J. Neurochem. 14: 873–885. 6051420 10.1111/j.1471-4159.1967.tb09576.x

[26] Spanner S., and Ansell G. B. 1978 The release of free ethanolamine in rat brain homogenates incubated in Krebs ringer. Adv. Exp. Med. Biol. 101: 247–251. 665367 10.1007/978-1-4615-9071-2_24

[27] Cotman C., Blank M. L., Moehl A., and Snyder F. 1969 Lipid composition of synaptic plasma membranes isolated from rat brain by zonal centrifugation. Biochemistry. 8: 4606–4612. 4311035 10.1021/bi00839a056

[28] Cotman C. W., McCaman R. E., and Dewhurst S. A. 1971 Subsynaptosomal distribution of enzymes involved in the metabolism of lipids. Biochim. Biophys. Acta. 249: 395–405. 4332410 10.1016/0005-2736(71)90118-0

[29] Palmer E., Monaghan D. T., and Cotman C. W. 1988 Glutamate receptors and phosphoinositide metabolism: stimulation via quisqualate receptors is inhibited by *N*-methyl-D-aspartate receptor activation. Brain Res. 464: 161–165. 2905924 10.1016/0169-328x(88)90008-3

[30] Palmer E., Nangel-Taylor K., Krause J. D., Roxas A., and Cotman C. W. 1990 Changes in excitatory amino acid modulation of phosphoinositide metabolism during development. Brain Res. Dev. Brain Res. 51: 132–134. 2153480 10.1016/0165-3806(90)90266-2

[31] Robinson B. S., Johnson D. W., and Poulos A. 1990 Metabolism of hexacosatetraenoic acid (C26:4n-6) in immature rat brain. Biochem. J. 267: 561–564. 2185745 10.1042/bj2670561 PMC1131329

[32] Robinson B. S., Johnson D. W., and Poulos A. 1990 Unique molecular species of phosphatidylcholine containing very-long-chain (C24–C38) polyenoic fatty acids in rat brain. Biochem. J. 265: 763–767. 2306213 10.1042/bj2650763 PMC1133699

[33] Johnson D. W., Beckman K., Fellenberg A. J., Robinson B. S., and Poulos A. 1992 Monoenoic fatty acids in human brain lipids: isomer identification and distribution. Lipids. 27: 177–180. 1522761 10.1007/BF02536174

[34] Poulos A. 1995 Very long chain fatty acids in higher animals—a review. Lipids. 30: 1–14. 7760683 10.1007/BF02537036

[35] O’Brien J. S., and Rouser G. 1964 The fatty acid composition of brain sphingolipids: sphingomyelin, ceramide, cerebroside, and cerebroside sulfate. J. Lipid Res. 5: 339–342. 5873370

[36] Siakotos A. N., Rouser G., and Fleischer S. 1969 Phospholipid composition of human, bovine and frog myelin isolated on a large scale from brain and spinal cord. Lipids. 4: 239–242. 4306678 10.1007/BF02532639

[37] Rouser G., Kritchevsky G., Yamamoto A., and Baxter C. F. 1972 Lipids in the nervous system of different species as a function of age: brain, spinal cord, peripheral nerve, purified whole cell preparations, and subcellular particulates: regulatory mechanisms and membrane structure. Adv. Lipid Res. 10: 261–360. 4344800 10.1016/b978-0-12-024910-7.50013-0

[38] Rouser G., and Yamamoto A. 1972 Ceramide, triglyceride, and sterol ester in normal human whole brain at different ages. Lipids. 7: 561–563. 5066424 10.1007/BF02533026

[39] Rouser G., and Yamamoto A. 1972 The fatty acid composition of total gangliosides in normal human whole brain at different ages. J. Neurochem. 19: 2697–2698. 5086252 10.1111/j.1471-4159.1972.tb01329.x

[40] Yamamoto A., and Rouser G. 1973 Free fatty acids of normal human whole brain at different ages. J. Gerontol. 28: 140–142. 4692310 10.1093/geronj/28.2.140

[41] Fraser T., Tayler H., and Love S. 2010 Fatty acid composition of frontal, temporal and parietal neocortex in the normal human brain and in Alzheimer’s disease. Neurochem. Res. 35: 503–513. 19904605 10.1007/s11064-009-0087-5

[42] Cunnane S. C., Schneider J. A., Tangney C., Tremblay-Mercier J., Fortier M., Bennett D. A., and Morris M. C. 2012 Plasma and brain fatty acid profiles in mild cognitive impairment and Alzheimer’s disease. J. Alzheimers Dis. 29: 691–697. 22466064 10.3233/JAD-2012-110629 PMC3409580

[43] Söderberg M., Edlund C., Kristensson K., and Dallner G. 1991 Fatty acid composition of brain phospholipids in aging and in Alzheimer’s disease. Lipids. 26: 421–425. 1881238 10.1007/BF02536067

[44] Kumar A., and Foster T. C. 2007 Neurophysiology of old neurons and synapses. *In* Riddle D. R., editor. Brain Aging: Models, Methods, and Mechanisms. Frontiers in Neuroscience. Frontiers, Boca Raton, FL.

[45] Long X., Liao W., Jiang C., Liang D., Qiu B., and Zhang L. 2012 Healthy aging: an automatic analysis of global and regional morphological alterations of human brain. Acad. Radiol. 19: 785–793. 22503890 10.1016/j.acra.2012.03.006

[46] Yoshiura T., Mihara F., Tanaka A., Togao O., Taniwaki T., Nakagawa A., Nakao T., Noguchi T., Kuwabara Y., and Honda H. 2005 Age-related structural changes in the young adult brain shown by magnetic resonance diffusion tensor imaging. Acad. Radiol. 12: 268–275. 15766685 10.1016/j.acra.2004.12.015

[47] Ginsberg L., Rafique S., Xuereb J. H., Rapoport S. I., and Gershfeld N. L. 1995 Disease and anatomic specificity of ethanolamine plasmalogen deficiency in Alzheimer’s disease brain. Brain Res. 698: 223–226. 8581486 10.1016/0006-8993(95)00931-f

[48] Hejazi L., Wong J. W., Cheng D., Proschogo N., Ebrahimi D., Garner B., and Don A. S. 2011 Mass and relative elution time profiling: two-dimensional analysis of sphingolipids in Alzheimer’s disease brains. Biochem. J. 438: 165–175. 21639855 10.1042/BJ20110566

[49] Martín V., Fabelo N., Santpere G., Puig B., Marin R., Ferrer I., and Diaz M. 2010 Lipid alterations in lipid rafts from Alzheimer’s disease human brain cortex. J. Alzheimers Dis. 19: 489–502. 20110596 10.3233/JAD-2010-1242

[50] Vanhooren V., and Libert C. 2013 The mouse as a model organism in aging research: usefulness, pitfalls and possibilities. Ageing Res. Rev. 12: 8–21. 22543101 10.1016/j.arr.2012.03.010

[51] Busik J. V., Reid G. E., and Lydic T. A. 2009 Global analysis of retina lipids by complementary precursor ion and neutral loss mode tandem mass spectrometry. Methods Mol. Biol. 579: 33–70. 19763470 10.1007/978-1-60761-322-0_3 PMC2879063

[52] Bligh E. G., and Dyer W. J. 1959 A rapid method of total lipid extraction and purification. Can. J. Biochem. Physiol. 37: 911–917. 13671378 10.1139/o59-099

[53] Martin R. E., Elliott M. H., Brush R. S., and Anderson R. E. 2005 Detailed characterization of the lipid composition of detergent-resistant membranes from photoreceptor rod outer segment membranes. Invest. Ophthalmol. Vis. Sci. 46: 1147–1154. 15790872 10.1167/iovs.04-1207

[54] Martin R. E. 1998 Docosahexaenoic acid decreases phospholipase A2 activity in the neurites/nerve growth cones of PC12 cells. J. Neurosci. Res. 54: 805–813. 9856864 10.1002/(SICI)1097-4547(19981215)54:6<805::AID-JNR8>3.0.CO;2-4

[55] Martin R. E., Hopkins S. A., Steven Brush R., Williamson C., Chen H., and Anderson R. E. 2002 Docosahexaenoic, arachidonic, palmitic, and oleic acids are differentially esterified into phospholipids of frog retina. Prostaglandins Leukot. Essent. Fatty Acids. 67: 105–111. 12324228 10.1054/plef.2002.0406

[56] Martin R. E., Wickham J. Q., Om A. S., Sanders J., and Ceballos N. 2000 Uptake and incorporation of docosahexaenoic acid (DHA) into neuronal cell body and neurite/nerve growth cone lipids: evidence of compartmental DHA metabolism in nerve growth factor-differentiated PC12 cells. Neurochem. Res. 25: 715–723. 10905634 10.1023/a:1007575406896

[57] Yu M., Benham A., Logan S., Brush R. S., Mandal M. N., Anderson R. E., and Agbaga M. P. 2012 ELOVL4 protein preferentially elongates 20:5n3 to very long chain PUFAs over 20:4n6 and 22:6n3. J. Lipid Res. 53: 494–504. 22158834 10.1194/jlr.M021386 PMC3276472

[58] Chalon S., Delion-Vancassel S., Belzung C., Guilloteau D., Leguisquet A. M., Besnard J. C., and Durand G. 1998 Dietary fish oil affects monoaminergic neurotransmission and behavior in rats. J. Nutr. 128: 2512–2519. 9868201 10.1093/jn/128.12.2512

[59] Crawford M. A., Broadhurst C. L., Guest M., Nagar A., Wang Y., Ghebremeskel K., and Schmidt W. F. 2013 A quantum theory for the irreplaceable role of docosahexaenoic acid in neural cell signalling throughout evolution. Prostaglandins Leukot. Essent. Fatty Acids. 88: 5–13. 23206328 10.1016/j.plefa.2012.08.005

[60] Scott B. L., and Bazan N. G. 1989 Membrane docosahexaenoate is supplied to the developing brain and retina by the liver. Proc. Natl. Acad. Sci. USA. 86: 2903–2907. 2523075 10.1073/pnas.86.8.2903 PMC287028

[61] Sergeeva M., Strokin M., and Reiser G. 2005 Regulation of intracellular calcium levels by polyunsaturated fatty acids, arachidonic acid and docosahexaenoic acid, in astrocytes: possible involvement of phospholipase A2. Reprod. Nutr. Dev. 45: 633–646. 16188212 10.1051/rnd:2005050

[62] Stillwell W., Shaikh S. R., Zerouga M., Siddiqui R., and Wassall S. R. 2005 Docosahexaenoic acid affects cell signaling by altering lipid rafts. Reprod. Nutr. Dev. 45: 559–579. 16188208 10.1051/rnd:2005046

[63] Stillwell W., and Wassall S. R. 2003 Docosahexaenoic acid: membrane properties of a unique fatty acid. Chem. Phys. Lipids. 126: 1–27. 14580707 10.1016/s0009-3084(03)00101-4

[64] Wassall S. R., and Stillwell W. 2008 Docosahexaenoic acid domains: the ultimate non-raft membrane domain. Chem. Phys. Lipids. 153: 57–63. 18343224 10.1016/j.chemphyslip.2008.02.010

[65] Bazan N. G. 2003 Synaptic lipid signaling: significance of polyunsaturated fatty acids and platelet-activating factor. J. Lipid Res. 44: 2221–2233. 13130128 10.1194/jlr.R300013-JLR200

[66] Labrousse V. F., Nadjar A., Joffre C., Costes L., Aubert A., Gregoire S., Bretillon L., and Laye S. 2012 Short-term long chain omega3 diet protects from neuroinflammatory processes and memory impairment in aged mice. PLoS One. 7: e36861. 22662127 10.1371/journal.pone.0036861 PMC3360741

[67] De Mel D., and Suphioglu C. 2014 Fishy business: effect of omega-3 fatty acids on zinc transporters and free zinc availability in human neuronal cells. Nutrients. 6: 3245–3258. 25195602 10.3390/nu6083245 PMC4145306

[68] Lauritzen L., Brambilla P., Mazzocchi A., Harslof L. B., Ciappolino V., and Agostoni C. 2016 DHA effects in brain development and function. Nutrients. 8: 6. 10.3390/nu8010006 PMC4728620 26742060

[69] Kim H. Y., Huang B. X., and Spector A. A. 2014 Phosphatidyl­serine in the brain: metabolism and function. Prog. Lipid Res. 56: 1–18. 24992464 10.1016/j.plipres.2014.06.002 PMC4258547

[70] Dratz E. A., Van Breemen J. F., Kamps K. M., Keegstra W., and Van Bruggen E. F. 1985 Two-dimensional crystallization of bovine rhodopsin. Biochim. Biophys. Acta. 832: 337–342. 4074754 10.1016/0167-4838(85)90268-7

[71] Hasadsri L., Wang B. H., Lee J. V., Erdman J. W., Llano D. A., Barbey A. K., Wszalek T., Sharrock M. F., and Wang H. J. 2013 Omega-3 fatty acids as a putative treatment for traumatic brain injury. J. Neurotrauma. 30: 897–906. 23363551 10.1089/neu.2012.2672

[72] Cansev M., and Wurtman R. J. 2007 Chronic administration of docosahexaenoic acid or eicosapentaenoic acid, but not arachidonic acid, alone or in combination with uridine, increases brain phosphatide and synaptic protein levels in gerbils. Neuroscience. 148: 421–431. 17683870 10.1016/j.neuroscience.2007.06.016 PMC2048660

[73] VanGuilder H. D., Farley J. A., Yan H., Van Kirk C. A., Mitschelen M., Sonntag W. E., and Freeman W. M. 2011 Hippocampal dysregulation of synaptic plasticity-associated proteins with age-related cognitive decline. Neurobiol. Dis. 43: 201–212. 21440628 10.1016/j.nbd.2011.03.012 PMC3096728

[74] VanGuilder H. D., Yan H., Farley J. A., Sonntag W. E., and Freeman W. M. 2010 Aging alters the expression of neurotransmission-regulating proteins in the hippocampal synaptoproteome. J. Neurochem. 113: 1577–1588. 20374424 10.1111/j.1471-4159.2010.06719.x PMC3010414

[75] Garcia M. C., Ward G., Ma Y. C., Salem N. Jr., and Kim H. Y. 1998 Effect of docosahexaenoic acid on the synthesis of phosphatidylserine in rat brain in microsomes and C6 glioma cells. J. Neurochem. 70: 24–30. 9422343 10.1046/j.1471-4159.1998.70010024.x

[76] Akbar M., Calderon F., Wen Z., and Kim H. Y. 2005 Docosa­hexaenoic acid: a positive modulator of Akt signaling in neuronal survival. Proc. Natl. Acad. Sci. USA. 102: 10858–10863. 16040805 10.1073/pnas.0502903102 PMC1182431

[77] Dyall S. C. 2015 Long-chain omega-3 fatty acids and the brain: a review of the independent and shared effects of EPA, DPA and DHA. Front. Aging Neurosci. 7: 52, 1–15. 25954194 10.3389/fnagi.2015.00052 PMC4404917

[78] Agbaga M. P., Brush R. S., Mandal M. N., Henry K., Elliott M. H., and Anderson R. E. 2008 Role of Stargardt-3 macular dystrophy protein (ELOVL4) in the biosynthesis of very long chain fatty acids. Proc. Natl. Acad. Sci. USA. 105: 12843–12848. 18728184 10.1073/pnas.0802607105 PMC2525561

[79] Edwards A. O., Donoso L. A., and Ritter R. 3rd 2001 A novel gene for autosomal dominant Stargardt-like macular dystrophy with homology to the SUR4 protein family. Invest. Ophthalmol. Vis. Sci. 42: 2652–2663. 11581213

[80] Ayyagari R., Zhang K., Hutchinson A., Yu Z., Swaroop A., Kakuk L. E., Seddon J. M., Bernstein P. S., Lewis R. A., Tammur J., 2001 Evaluation of the ELOVL4 gene in patients with age-related macular degeneration. Ophthalmic Genet. 22: 233–239. 11803489 10.1076/opge.22.4.233.2219

[81] Bernstein P. S., Tammur J., Singh N., Hutchinson A., Dixon M., Pappas C. M., Zabriskie N. A., Zhang K., Petrukhin K., Leppert M., 2001 Diverse macular dystrophy phenotype caused by a novel complex mutation in the ELOVL4 gene. Invest. Ophthalmol. Vis. Sci. 42: 3331–3336. 11726641

[82] Zhang K., Kniazeva M., Han M., Li W., Yu Z., Yang Z., Li Y., Metzker M. L., Allikmets R., Zack D. J., 2001 A 5-bp deletion in ELOVL4 is associated with two related forms of autosomal dominant macular dystrophy. Nat. Genet. 27: 89–93. 11138005 10.1038/83817

[83] Aldahmesh M. A., Mohamed J. Y., Alkuraya H. S., Verma I. C., Puri R. D., Alaiya A. A., Rizzo W. B., and Alkuraya F. S. 2011 Recessive mutations in ELOVL4 cause ichthyosis, intellectual disability, and spastic quadriplegia. Am. J. Hum. Genet. 89: 745–750. 22100072 10.1016/j.ajhg.2011.10.011 PMC3234380

[84] Cadieux-Dion M., Turcotte-Gauthier M., Noreau A., Martin C., Meloche C., Gravel M., Drouin C. A., Rouleau G. A., Nguyen D. K., and Cossette P. 2014 Expanding the clinical phenotype associated with ELOVL4 mutation: study of a large French-Canadian family with autosomal dominant spinocerebellar ataxia and erythrokeratodermia. JAMA Neurol. 71: 470–475. 24566826 10.1001/jamaneurol.2013.6337

[85] Mir H., Raza S. I., Touseef M., Memon M. M., Khan M. N., Jaffar S., and Ahmad W. 2014 A novel recessive mutation in the gene ELOVL4 causes a neuro-ichthyotic disorder with variable expressivity. BMC Med. Genet. 15: 25, 1–5. 24571530 10.1186/1471-2350-15-25 PMC3941482

[86] Bourassa C. V., Raskin S., Serafini S., Teive H. A., Dion P. A., and Rouleau G. A. 2015 A new ELOVL4 mutation in a case of spinocerebellar ataxia with erythrokeratodermia. JAMA Neurol. 72: 942–943. 26258735 10.1001/jamaneurol.2015.0888

[87] Ozaki K., Doi H., Mitsui J., Sato N., Iikuni Y., Majima T., Yamane K., Irioka T., Ishiura H., Doi K., 2015 A novel mutation in ELOVL4 leading to spinocerebellar ataxia (SCA) with the hot cross bun sign but lacking erythrokeratodermia: a broadened spectrum of SCA34. JAMA Neurol. 72: 797–805. 26010696 10.1001/jamaneurol.2015.0610

[88] Vasireddy V., Uchida Y., Salem N. Jr., Kim S. Y., Mandal M. N., Reddy G. B., Bodepudi R., Alderson N. L., Brown J. C., Hama H., 2007 Loss of functional ELOVL4 depletes very long-chain fatty acids (> or =C28) and the unique omega-O-acylceramides in skin leading to neonatal death. Hum. Mol. Genet. 16: 471–482. 17208947 10.1093/hmg/ddl480 PMC1839956

[89] McMahon A., Butovich I. A., and Kedzierski W. 2011 Epidermal expression of an Elovl4 transgene rescues neonatal lethality of homozygous Stargardt disease-3 mice. J. Lipid Res. 52: 1128–1138. 21429867 10.1194/jlr.M014415 PMC3090234

[90] Li W., Sandhoff R., Kono M., Zerfas P., Hoffmann V., Ding B. C., Proia R. L., and Deng C. X. 2007 Depletion of ceramides with very long chain fatty acids causes defective skin permeability barrier function, and neonatal lethality in ELOVL4 deficient mice. Int. J. Biol. Sci. 3: 120–128. 17311087 10.7150/ijbs.3.120 PMC1796950

[91] Cameron D. J., Tong Z., Yang Z., Kaminoh J., Kamiyah S., Chen H., Zeng J., Chen Y., Luo L., and Zhang K. 2007 Essential role of Elovl4 in very long chain fatty acid synthesis, skin permeability barrier function, and neonatal survival. Int. J. Biol. Sci. 3: 111–119. 17304340 10.7150/ijbs.3.111 PMC1796949

[92] Poulos A., Johnson D. W., Beckman K., White I. G., and Easton C. 1987 Occurrence of unusual molecular species of sphingomyelin containing 28–34-carbon polyenoic fatty acids in ram spermatozoa. Biochem. J. 248: 961–964. 3435495 10.1042/bj2480961 PMC1148644

[93] McMahon A., Lu H., and Butovich I. A. 2014 A role for ELOVL4 in the mouse meibomian gland and sebocyte cell biology. Invest. Ophthalmol. Vis. Sci. 55: 2832–2840. 24677106 10.1167/iovs.13-13335 PMC4008046

[94] Aveldaño M. I., and Sprecher H. 1987 Very long chain (C24 to C36) polyenoic fatty acids of the n-3 and n-6 series in dipolyunsaturated phosphatidylcholines from bovine retina. J. Biol. Chem. 262: 1180–1186. 3805016

[95] Mandal M. N., Ambasudhan R., Wong P. W., Gage P. J., Sieving P. A., and Ayyagari R. 2004 Characterization of mouse orthologue of ELOVL4: genomic organization and spatial and temporal expression. Genomics. 83: 626–635. 15028285 10.1016/j.ygeno.2003.09.020

[96] Bazan N. G., Musto A. E., and Knott E. J. 2011 Endogenous signaling by omega-3 docosahexaenoic acid-derived mediators sustains homeostatic synaptic and circuitry integrity. Mol. Neurobiol. 44: 216–222. 21918832 10.1007/s12035-011-8200-6 PMC3180614

[97] Arnoldussen I. A., and Kiliaan A. J. 2014 Impact of DHA on metabolic diseases from womb to tomb. Mar. Drugs. 12: 6190–6212. 25528960 10.3390/md12126190 PMC4278225

[98] Debuch H. 1958 Nature of the linkage of the aldehyde residue of natural plasmalogens. J. Neurochem. 2: 243–248. 13576152 10.1111/j.1471-4159.1958.tb12370.x

[99] Rapport M. M., Lerner B., Alonzo N., and Franzl R. E. 1957 The structure of plasmalogens. II. Crystalline lysophosphatidal ethanolamine (acetal phospholipide). J. Biol. Chem. 225: 859–867. 13416288

[100] Marinetti G. V., Erbland J., Kochen J., and Stotz E. 1958 The phosphatide composition of a purified cytochrome oxidase preparation. J. Biol. Chem. 233: 740–742. 13575448

[101] Zoeller R. A., Morand O. H., and Raetz C. R. 1988 A possible role for plasmalogens in protecting animal cells against photosensitized killing. J. Biol. Chem. 263: 11590–11596. 3403547

[102] Nagan N., Hajra A. K., Larkins L. K., Lazarow P., Purdue P. E., Rizzo W. B., and Zoeller R. A. 1998 Isolation of a Chinese hamster fibroblast variant defective in dihydroxyacetonephosphate acyltransferase activity and plasmalogen biosynthesis: use of a novel two-step selection protocol. Biochem. J. 332: 273–279. 9576878 10.1042/bj3320273 PMC1219478

[103] Hagar H., Ueda N., and Shah S. V. 1996 Role of reactive oxygen metabolites in DNA damage and cell death in chemical hypoxic injury to LLC-PK1 cells. Am. J. Physiol. 271: F209–F215. 8760263 10.1152/ajprenal.1996.271.1.F209

[104] Dawson T. L., Gores G. J., Nieminen A. L., Herman B., and Lemasters J. J. 1993 Mitochondria as a source of reactive oxygen species during reductive stress in rat hepatocytes. Am. J. Physiol. 264: C961–C967. 8386454 10.1152/ajpcell.1993.264.4.C961

[105] Engelmann B., Brautigam C., and Thiery J. 1994 Plasmalogen phospholipids as potential protectors against lipid peroxidation of low density lipoproteins. Biochem. Biophys. Res. Commun. 204: 1235–1242. 7980601 10.1006/bbrc.1994.2595

[106] Jürgens G., Fell A., Ledinski G., Chen Q., and Paltauf F. 1995 Delay of copper-catalyzed oxidation of low density lipoprotein by in vitro enrichment with choline or ethanolamine plasmalogens. Chem. Phys. Lipids. 77: 25–31. 7586089 10.1016/0009-3084(95)02451-n

[107] Reiss D., Beyer K., and Engelmann B. 1997 Delayed oxidative degradation of polyunsaturated diacyl phospholipids in the presence of plasmalogen phospholipids in vitro. Biochem. J. 323: 807–814. 9169616 10.1042/bj3230807 PMC1218386

[108] Hahnel D., Beyer K., and Engelmann B. 1999 Inhibition of peroxyl radical-mediated lipid oxidation by plasmalogen phospholipids and alpha-tocopherol. Free Radic. Biol. Med. 27: 1087–1094. 10569641 10.1016/s0891-5849(99)00142-2

[109] Heymans H. S., Schutgens R. B., Tan R., van den Bosch H., and Borst P. 1983 Severe plasmalogen deficiency in tissues of infants without peroxisomes (Zellweger syndrome). Nature. 306: 69–70. 6633659 10.1038/306069a0

[110] Glaser P. E., and Gross R. W. 1994 Plasmenylethanolamine facilitates rapid membrane fusion: a stopped-flow kinetic investigation correlating the propensity of a major plasma membrane constituent to adopt an HII phase with its ability to promote membrane fusion. Biochemistry. 33: 5805–5812. 8180209 10.1021/bi00185a019

[111] Colley P. A., and Routtenberg A. 1993 Long-term potentiation as synaptic dialogue. Brain Res. Brain Res. Rev. 18: 115–122. 8467347 10.1016/0165-0173(93)90009-o

[112] Klann E., Chen S. J., and Sweatt J. D. 1991 Persistent protein kinase activation in the maintenance phase of long-term potentiation. J. Biol. Chem. 266: 24253–24256. 1684790

[113] Klann E., Chen S. J., and Sweatt J. D. 1993 Mechanism of protein kinase C activation during the induction and maintenance of long-term potentiation probed using a selective peptide substrate. Proc. Natl. Acad. Sci. USA. 90: 8337–8341. 8378303 10.1073/pnas.90.18.8337 PMC47351

[114] Leahy J. C., Luo Y., Kent C. S., Meiri K. F., and Vallano M. L. 1993 Demonstration of presynaptic protein kinase C activation following long-term potentiation in rat hippocampal slices. Neuroscience. 52: 563–574. 8095708 10.1016/0306-4522(93)90406-6

[115] Lovinger D. M., and Routtenberg A. 1988 Synapse-specific protein kinase C activation enhances maintenance of long-term potentiation in rat hippocampus. J. Physiol. 400: 321–333. 3418528 10.1113/jphysiol.1988.sp017122 PMC1191809

[116] Wang J. H., and Feng D. P. 1992 Postsynaptic protein kinase C essential to induction and maintenance of long-term potentiation in the hippocampal CA1 region. Proc. Natl. Acad. Sci. USA. 89: 2576–2580. 1557361 10.1073/pnas.89.7.2576 PMC48704

[117] Ganong B. R., Loomis C. R., Hannun Y. A., and Bell R. M. 1986 Specificity and mechanism of protein kinase C activation by sn-1,2-diacylglycerols. Proc. Natl. Acad. Sci. USA. 83: 1184–1188. 3456578 10.1073/pnas.83.5.1184 PMC323039

[118] Ford D. A., Miyake R., Glaser P. E., and Gross R. W. 1989 Activation of protein kinase C by naturally occurring ether-linked diglycerides. J. Biol. Chem. 264: 13818–13824. 2760045

[119] Mandal A., Wang Y., Ernsberger P., and Kester M. 1997 Interleukin-1-induced ether-linked diglycerides inhibit calcium-insensitive protein kinase C isotypes. Implications for growth senescence. J. Biol. Chem. 272: 20306–20311. 9242712 10.1074/jbc.272.32.20306

[120] Li X. W., Cao L., Wang F., Yang Q. G., Tong J. J., Li X. Y., and Chen G. H. 2016 Maternal inflammation linearly exacerbates offspring age-related changes of spatial learning and memory, and neurobiology until senectitude. Behav. Brain Res. 306: 178–196. 26992827 10.1016/j.bbr.2016.03.011

[121] Reagh Z. M., Ho H. D., Leal S. L., Noche J. A., Chun A., Murray E. A., and Yassa M. A. 2016 Greater loss of object than spatial mnemonic discrimination in aged adults. Hippocampus. 26: 417–422. 26691235 10.1002/hipo.22562 PMC5918289

[122] Soutif-Veillon A., Ferland G., Rolland Y., Presse N., Boucher K., Feart C., and Annweiler C. 2016 Increased dietary vitamin K intake is associated with less severe subjective memory complaint among older adults. Maturitas 93: 131–136. 26923488 10.1016/j.maturitas.2016.02.004

[123] Sung J. E. 2016 Age-related decline in case-marker processing and its relation to working memory Capacity. J. Gerontol. B Psychol. Sci. Soc. Sci. Epub ahead of print. January 16, 2016; doi:. 10.1093/geronb/gbv117 26773313

[124] Varga A. W., Ducca E. L., Kishi A., Fischer E., Parekh A., Koushyk V., Yau P. L., Gumb T., Leibert D. P., Wohlleber M. E., 2016 Effects of aging on slow-wave sleep dynamics and human spatial navigational memory consolidation. Neurobiol. Aging. 42: 142–149. 27143431 10.1016/j.neurobiolaging.2016.03.008 PMC4857208

[125] Zoeller R. A., Grazia T. J., LaCamera P., Park J., Gaposchkin D. P., and Farber H. W. 2002 Increasing plasmalogen levels protects human endothelial cells during hypoxia. Am. J. Physiol. Heart Circ. Physiol. 283: H671–H679. 12124215 10.1152/ajpheart.00524.2001

[126] Piomelli D., Astarita G., and Rapaka R. 2007 A neuroscientist’s guide to lipidomics. Nat. Rev. Neurosci. 8: 743–754. 17882252 10.1038/nrn2233

