# Supplementary material for: Regional changes in CNS and retinal glycerophospholipid profiles with age: a molecular blueprint
Source: J Lipid Res. 2017 Mar 29;58(4):668–80. doi: 10.1194/jlr.M070714 (PMC5392743; doi:10.1194/jlr.M070714)
Supplement: Supplemental Data [file supp_58_4_668__index.html]

Regional Changes in CNS and Retinal Glycerophospholipid Profiles with Age - A Molecular Blueprint — Regional changes in CNS and retinal glycerophospholipid profiles with age: a molecular blueprint — Supplemental Data 

# Regional changes in CNS and retinal glycerophospholipid profiles with age: a molecular blueprint

## Supplemental Data

- Supplemental Material (.pdf, 1.2 MB) - Supplemental Material
