# Supplementary material for: Regional changes in CNS and retinal glycerophospholipid profiles with age: a molecular blueprint
Source: J Lipid Res. 2017 Mar 29;58(4):668–80. doi: 10.1194/jlr.M070714 (PMC5392743; doi:10.1194/jlr.M070714)
Supplement: Supplemental Data [file 10.1194_M070714_jlr.M070714-1.pdf]

SUPPLEMENTARY INFORMATION:

Regional Changes in CNS and Retinal Glycerophospholipid Profiles with Age – A Molecular Blueprint

Blake R. Hopiavuori[1], Martin-Paul Agbaga[1,2,4,5], Richard S. Brush[2,5], Michael T. Sullivan[2], William E. Sonntag[1,3], and Robert E. Anderson[1,2,4,5,*]

1 Oklahoma Center for Neuroscience, University of Oklahoma Health Sciences Center, 975 N.E. 10th Street, BRC 272, Oklahoma City, OK 73104

2 Department of Ophthalmology, University of Oklahoma Health Sciences Center, 608 Stanton L. Young Blvd, Oklahoma City, OK 73104

3 Department of Geriatric Medicine, University of Oklahoma Health Sciences Center, 975 N.E. 10th Street, BRC 1303, Oklahoma City, OK 73104

4 Department of Cell Biology, University of Oklahoma Health Sciences Center, 940 Stanton L. Young Blvd, BMS 553, Oklahoma City, OK 73104

5 Dean McGee Eye Institute, 608 Stanton L. Young Blvd, Oklahoma City, OK 73104

Supplementary Table S1: Molecular Species Composition of Retinal Phosphatidylcholine

PC Molecular Species	% at 2 mo.	% at 10 mo.	% at 26 mo.	2 vs. 10 mo.	2 vs. 26 mo.	10 vs. 26 mo.
**30:00**[1]	0.69	0.54	0.65	ns	ns	ns
**30:01**	0.24	0.42	0.53	ns	ns	ns
**32:00**	17.23	15.43	15.02	$p < 0.0001$	$p < 0.0001$	ns
**32:01**	1.88	1.98	2.07	ns	ns	ns
**32:02**	0.27	0.44	0.50	ns	ns	ns
**34:00**	4.68	4.22	4.18	ns	ns	ns
**34:01**	17.91	18.80	18.24	$p = 0.0008$	ns	ns
**34:02**	1.92	2.31	2.05	ns	ns	ns
**36:01**	7.59	7.62	7.62	ns	ns	ns
**36:02**	2.10	2.33	2.21	ns	ns	ns
**36:03**	0.84	0.94	0.80	ns	ns	ns
**36:04**	1.86	2.38	2.33	ns	ns	ns
**38:02**	0.31	0.38	0.39	ns	ns	ns
**38:03**	0.52	0.52	0.49	ns	ns	ns
**38:04**	3.09	3.28	3.28	ns	ns	ns
**38:05**	0.81	0.85	0.86	ns	ns	ns
**38:06**	11.48	10.88	10.59	$p = 0.0353$	$p = 0.0007$	ns
**40:06**	15.72	15.90	16.88	ns	$p < 0.0001$	$p = 0.0002$
**40:07**	1.21	1.14	1.21	ns	ns	ns
**44:12**	3.77	3.73	4.13	ns	ns	ns
**46:12**	0.30	0.27	0.30	ns	ns	ns
**Σ VLC-PUFA**	4.09	4.11	3.91	ns	ns	ns

1-The nomenclature for molecular species is as follows: 30:00 has one molecule of 14:0 and one molecule of 16:0 (14:0/16:0).40:06 contains one molecule of 18:0 and one molecule of 22:6 (18:0/22:6).

Supplementary *Table S2: Molecular Species Composition of Retinal Phosphatidylethanolamine*

PE Molecular Species	% at 2 mo.	% at 10 mo.	% at 26 mo.	2 vs. 10 mo.	2 vs. 26 mo.	10 vs. 26 mo.
**34:00**	0.32	0.22	0.26	ns	ns	ns
**34:01**	1.31	1.36	1.47	ns	ns	ns
**34:02**	0.31	0.34	0.50	ns	ns	ns
**36:00p**[1]	0.33	0.36	0.37	ns	ns	ns
**36:01**	1.49	1.53	1.68	ns	ns	ns
**36:02**	0.62	0.93	1.15	ns	ns	ns
**36:03**	0.35	0.42	0.31	ns	ns	ns
**36:04**	1.06	1.38	1.31	ns	ns	ns
**38:00**	0.64	0.52	0.55	ns	ns	ns
**38:03**	0.52	0.44	0.44	ns	ns	ns
**38:04**	5.58	5.88	6.26	ns	$p = 0.0229$	ns
**38:04p**	0.24	0.42	0.42	ns	ns	ns
**38:05**	1.47	1.46	1.61	ns	ns	ns
**38:05p**	1.06	0.91	0.84	ns	ns	ns
**38:06**	22.82	21.67	19.07	$p < 0.0001$	$p < 0.0001$	$p < 0.0001$
**40:04**	0.78	0.67	0.72	ns	ns	ns
**40:05**	0.74	0.71	0.67	ns	ns	ns
**40:05p**	1.29	1.16	1.25	ns	ns	ns
**40:06**	39.33	38.19	38.39	$p < 0.0001$	$p = 0.0009$	ns
**40:07**	4.47	4.25	3.93	ns	ns	ns
**40:08**	1.00	0.81	0.47	ns	ns	ns
**40:09**	0.26	0.23	0.20	ns	ns	ns
**42:06**	0.16	0.19	0.21	ns	ns	ns
**42:07**	0.29	0.26	0.19	ns	ns	ns
**42:08**	0.49	0.44	0.38	ns	ns	ns
**42:10**	0.47	0.40	0.39	ns	ns	ns
**44:10**	0.40	0.34	0.40	ns	ns	ns
**44:11**	0.72	0.84	0.81	ns	ns	ns
**44:12**	9.37	10.16	11.25	$p = 0.0063$	$p < 0.0001$	$p < 0.0001$
**46:11**	0.24	0.29	0.24	ns	ns	ns
**46:12**	0.52	0.51	0.54	ns	ns	ns
**Σ Ether PE**	2.59	3.39	3.65	$p = 0.006$	$p = 0.0001$	ns

1- p identifies plasmalogens (Ether PE).

Supplementary *Table S3: Molecular Species Composition of Retinal Phosphatidylserine*

PS Molecular Species	% at 2 mo.	% at 10 mo.	% at 26 mo.	2 vs. 10 mo.	2 vs. 26 mo.	10 vs. 26 mo.
**34:01**	0.42	0.42	0.82	ns	ns	ns
**36:00**	1.15	0.86	0.78	ns	ns	ns
**36:01**	0.94	3.02	3.06	$p = 0.0042$	$p = 0.0034$	ns
**36:02**	0.74	1.50	2.25	ns	ns	ns
**38:04**	7.68	9.93	10.88	$p = 0.0018$	$p < 0.0001$	ns
**38:05**	0.59	0.93	1.03	ns	ns	ns
**38:06**	0.28	0.32	0.47	ns	ns	ns
**40:04**	2.34	1.80	2.20	ns	ns	ns
**40:05**	0.74	0.74	0.92	ns	ns	ns
**40:06**	42.70	37.86	38.28	$p < 0.0001$	$p < 0.0001$	ns
**40:07**	0.72	0.70	0.77	ns	ns	ns
**42:06**	0.36	0.42	0.26	ns	ns	ns
**42:07**	0.25	0.15	0.21	ns	ns	ns
**42:09**	0.59	0.57	0.50	ns	ns	ns
**44:11**	3.26	3.89	3.83	ns	ns	ns
**44:12**	20.44	20.90	19.28	ns	ns	$p = 0.0345$
**46:11**	3.84	4.41	3.40	ns	ns	ns
**46:12**	8.29	7.71	6.86	ns	ns	ns
**48:12**	0.62	0.52	0.46	ns	ns	ns

Supplementary *Table S4: Molecular Species Composition of Hippocampal Phosphatidylcholine*

PC Molecular Species	% at 2 mo.	% at 10 mo.	% at 26 mo.	2 vs. 10 mo.	2 vs. 26 mo.	10 vs. 26 mo.
**32:00**	22.78	26.24	26.06	$p < 0.0001$	$p < 0.0001$	ns
**32:00e**	0.38	0.37	0.31	ns	ns	ns
**32:01**	2.58	3.87	4.40	$p < 0.0001$	$p < 0.0001$	$p = 0.0021$
**32:02**	1.38	2.47	3.07	$p < 0.0001$	$p < 0.0001$	$p = 0.0005$
**34:00**	5.44	4.78	4.63	$p < 0.0001$	$p < 0.0001$	ns
**34:00p**	0.62	0.53	0.49	ns	ns	ns
**34:01**	32.60	29.74	29.87	$p < 0.0001$	$p < 0.0001$	ns
**34:02**	1.13	1.01	1.00	ns	ns	ns
**36:01**	9.17	8.44	8.71	$p < 0.0001$	$p = 0.0104$	ns
**36:02**	2.61	2.17	2.24	$p = 0.0135$	$p = 0.0472$	ns
**36:03**	0.70	0.60	0.49	ns	ns	ns
**36:04**	5.61	5.14	4.77	$p = 0.0066$	$p < 0.0001$	ns
**38:01**	0.33	0.28	0.26	ns	ns	ns
**38:02**	0.48	0.43	0.48	ns	ns	ns
**38:03**	0.38	0.42	0.45	ns	ns	ns
**38:04**	5.96	5.43	4.89	$p = 0.0025$	$p < 0.0001$	$p = 0.002$
**38:05**	1.62	1.56	1.39	ns	ns	ns
**38:06**	3.65	3.43	3.48	ns	ns	ns
**40:06**	1.66	1.56	1.54	ns	ns	ns
**40:07**	0.58	0.58	0.55	ns	ns	ns

Supplementary *Table S5: Molecular Species Composition of Hippocampal Phosphatidylethanolamine*

PE Molecular Species	% at 2 mo.	% at 10 mo.	% at 26 mo.	2 vs. 10 mo.	2 vs. 26 mo.	10 vs. 26 mo.
**34:00**	0.29	0.67	0.67	ns	ns	ns
**34:01**	3.01	3.51	4.87	ns	$p < 0.0001$	$p < 0.0001$
**36:00**	0.64	0.44	0.50	ns	ns	ns
**36:01**	5.08	6.21	7.47	$p < 0.0001$	$p < 0.0001$	$p < 0.0001$
**36:02**	3.77	4.09	4.67	ns	$p = 0.0006$	$p = 0.0394$
**36:03**	0.43	0.57	0.49	ns	ns	ns
**36:04**	2.77	2.64	3.05	ns	ns	ns
**38:00**	1.38	0.98	0.88	ns	ns	ns
**38:01**	0.62	0.59	0.61	ns	ns	ns
**38:02**	0.35	0.34	0.44	ns	ns	ns
**38:03**	0.34	0.64	0.72	ns	ns	ns
**38:03p**	0.70	0.54	0.54	ns	ns	ns
**38:04**	25.55	21.95	20.93	$p < 0.0001$	$p < 0.0001$	$p < 0.0001$
**38:04p**	0.69	0.47	0.50	ns	ns	ns
**38:05**	3.12	2.94	3.03	ns	ns	ns
**38:05p**	1.21	1.24	1.10	ns	ns	ns
**38:06**	10.98	9.84	9.09	$p < 0.0001$	$p < 0.0001$	$p = 0.0054$
**40:04**	3.24	3.40	3.25	ns	ns	ns
**40:05**	0.78	1.09	1.05	ns	ns	ns
**40:05p**	1.24	0.89	0.89	ns	ns	ns
**40:06**	31.13	29.81	27.84	$p < 0.0001$	$p < 0.0001$	$p < 0.0001$
**40:07**	2.68	2.73	2.53	ns	ns	ns
**Σ Ether PE**	3.84	3.13	3.03	$p = 0.0099$	$p = 0.0023$	ns

Supplementary *Table S6: Molecular Species Composition of Hippocampal Phosphatidylserine*

PS Molecular Species	% at 2 mo.	% at 10 mo.	% at 26 mo.	2 vs. 10 mo.	2 vs. 26 mo.	10 vs. 26 mo.
**34:01**	2.13	1.06	1.26	ns	ns	ns
**36:01**	12.28	16.00	21.95	$p = 0.0002$	$p < 0.0001$	$p < 0.0001$
**36:02**	3.99	3.29	3.13	ns	ns	ns
**38:01**	0.49	0.98	1.76	ns	ns	ns
**38:04**	2.51	2.44	3.93	ns	ns	ns
**40:01**	1.21	0.56	1.12	ns	ns	ns
**40:04**	4.78	5.56	3.88	ns	ns	ns
**40:06**	63.29	64.22	56.13	ns	$p < 0.0001$	$p < 0.0001$
**40:07**	0.54	0.82	0.78	ns	ns	ns
**42:01**	0.37	0.21	0.35	ns	ns	ns
**44:10**	0.34	0.88	0.58	ns	ns	ns
**44:12**	1.04	0.98	0.33	ns	ns	ns

Supplementary *Table S7: Molecular Species Composition of Cerebellar Phosphatidylcholine*

PC Molecular Species	% at 2 mo.	% at 10 mo.	% at 26 mo.	2 vs. 10 mo.	2 vs. 26 mo.	10 vs. 26 mo.
**32:00**	12.09	13.65	12.71	$p < 0.0001$	$p = 0.0002$	$p < 0.0001$
**32:01**	1.54	1.74	1.81	ns	ns	ns
**32:02**	0.27	0.65	0.64	$p = 0.0353$	$p = 0.0402$	ns
**34:00**	5.22	5.27	4.49	ns	$p < 0.0001$	$p < 0.0001$
**34:01**	32.02	31.97	33.03	ns	$p < 0.0001$	$p < 0.0001$
**34:02**	1.59	1.41	1.38	ns	ns	ns
**36:01**	11.47	12.42	11.96	$p < 0.0001$	$p = 0.0047$	$p = 0.0083$
**36:02**	3.46	3.39	3.58	ns	ns	ns
**36:03**	0.73	0.61	0.62	ns	ns	ns
**36:04**	3.34	2.90	3.35	$p = 0.0137$	ns	$p = 0.0119$
**38:01**	0.98	0.97	0.85	ns	ns	ns
**38:02**	1.01	1.00	0.99	ns	ns	ns
**38:03**	0.64	0.60	0.57	ns	ns	ns
**38:04**	3.54	3.09	3.20	$p = 0.0117$	ns	ns
**38:05**	1.29	1.16	1.27	ns	ns	ns
**38:06**	8.24	7.97	8.53	ns	ns	$p = 0.0011$
**40:06**	8.35	7.66	7.26	$p < 0.0001$	$p < 0.0001$	$p = 0.0304$
**40:07**	2.02	1.93	2.02	ns	ns	ns
**42:07**	0.36	0.33	0.32	ns	ns	ns
**44:12**	0.44	0.43	0.49	ns	ns	ns

Supplementary *Table S8: Molecular Species Composition of Cerebellar Phosphatidylethanolamine*

PE Molecular Species	% at 2 mo.	% at 10 mo.	% at 26 mo.	2 vs. 10 mo.	2 vs. 26 mo.	10 vs. 26 mo.
**32:00**	0.14	0.17	0.16	ns	ns	ns
**32:01**	0.11	0.09	0.13	ns	ns	ns
**34:00**	0.36	0.46	0.39	ns	ns	ns
**34:00p**	0.40	0.62	0.57	ns	ns	ns
**34:01**	4.57	5.44	5.14	$p < 0.0001$	$p < 0.0001$	$p = 0.0183$
**34:01p**	0.38	0.34	0.37	ns	ns	ns
**34:02**	0.69	0.57	0.59	ns	ns	ns
**36:00**	0.32	0.32	0.34	ns	ns	ns
**36:00p**	0.35	0.53	0.57	ns	ns	ns
**36:01**	4.79	6.87	7.20	$p < 0.0001$	$p < 0.0001$	$p = 0.0097$
**36:01p**	0.76	0.97	0.89	ns	ns	ns
**36:02**	7.65	8.30	8.38	$p < 0.0001$	$p < 0.0001$	ns
**36:02p**	0.75	1.12	1.27	$p = 0.0025$	$p < 0.0001$	ns
**36:03**	0.74	0.73	0.68	ns	ns	ns
**36:04**	1.65	1.70	2.04	ns	$p = 0.0013$	$p = 0.006$
**36:05**	0.18	0.15	0.17	ns	ns	ns
**38:00**	1.19	1.03	0.93	ns	ns	ns
**38:00e**	0.11	0.14	0.15	ns	ns	ns
**38:00p**	0.19	0.24	0.27	ns	ns	ns
**38:01**	0.92	1.09	0.99	ns	ns	ns
**38:01p**	0.36	0.45	0.47	ns	ns	ns
**38:02**	1.06	1.12	1.12	ns	ns	ns
**38:02p**	0.31	0.45	0.59	ns	$p = 0.0364$	ns
**38:03**	0.66	0.55	0.54	ns	ns	ns
**38:03p**	0.36	0.35	0.36	ns	ns	ns
**38:04**	11.34	10.78	11.36	$p < 0.0001$	ns	$p < 0.0001$
**38:04p**	0.47	0.41	0.50	ns	ns	ns
**38:05**	4.14	3.96	4.72	ns	$p < 0.0001$	$p < 0.0001$
**38:05p**	0.73	0.77	0.86	ns	ns	ns
**38:06**	9.76	9.02	8.72	$p < 0.0001$	$p < 0.0001$	$p = 0.02$
**40:02**	0.15	0.17	0.23	ns	ns	ns
**40:03**	0.08	0.14	0.12	ns	ns	ns
**40:03p**	0.13	0.14	0.13	ns	ns	ns
**40:04**	1.11	1.17	1.18	ns	ns	ns
**40:04p**	0.22	0.23	0.26	ns	ns	ns
**40:05**	0.76	0.73	0.71	ns	ns	ns
**40:05p**	1.15	1.04	1.01	ns	ns	ns
**40:06**	33.45	30.05	27.70	$p < 0.0001$	$p < 0.0001$	$p < 0.0001$
**40:07**	4.04	3.83	3.96	ns	ns	ns
**40:08**	0.34	0.30	0.37	ns	ns	ns
**40:09**	0.13	0.23	0.19	ns	ns	ns
**42:07**	0.26	0.25	0.26	ns	ns	ns
**42:08**	0.13	0.13	0.12	ns	ns	ns
**42:10**	0.24	0.23	0.28	ns	ns	ns
**44:10**	0.62	0.51	0.52	ns	ns	ns
**44:11**	0.14	0.13	0.15	ns	ns	ns
**44:12**	1.01	1.00	1.05	ns	ns	ns
**Σ Ether PE**	6.67	7.81	8.27	$p < 0.0001$	$p < 0.0001$	$p = 0.0002$

Supplementary *Table S9: Molecular Species Composition of Cerebellar Phosphatidylserine*

PS Molecular Species	% at 2 mo.	% at 10 mo.	% at 26 mo.	2 vs. 10 mo.	2 vs. 26 mo.	10 vs. 26 mo.
**34:01**	1.58	1.44	1.36	ns	ns	ns
**36:01**	21.43	31.54	33.92	$p < 0.0001$	$p < 0.0001$	$p < 0.0001$
**36:02**	14.70	12.84	11.81	$p < 0.0001$	$p < 0.0001$	$p = 0.0159$
**38:01**	2.99	3.94	3.77	$p = 0.016$	ns	ns
**38:02**	1.41	1.41	1.56	ns	ns	ns
**38:04**	3.13	2.92	3.60	ns	ns	ns
**38:05**	0.72	0.57	0.50	ns	ns	ns
**38:06**	0.37	0.25	0.23	ns	ns	ns
**38:08**	0.20	0.36	0.47	ns	ns	ns
**40:01**	1.03	1.39	1.42	ns	ns	ns
**40:02**	0.72	0.95	0.91	ns	ns	ns
**40:04**	1.83	1.69	1.98	ns	ns	ns
**40:06**	40.94	32.60	29.91	$p < 0.0001$	$p < 0.0001$	$p < 0.0001$
**40:07**	1.40	1.07	1.09	ns	ns	ns
**40:08**	0.29	0.35	0.46	ns	ns	ns
**42:01**	0.56	0.64	0.70	ns	ns	ns
**42:02**	0.39	0.57	0.64	ns	ns	ns
**42:07**	0.44	0.30	0.30	ns	ns	ns
**42:08**	0.33	0.46	0.51	ns	ns	ns
**42:09**	0.63	1.19	1.08	ns	ns	ns
**44:10**	1.19	0.78	0.87	ns	ns	ns
**44:12**	2.93	2.12	2.21	$p = 0.0467$	ns	ns

Supplementary *Table S10: Molecular Species Composition of Brainstem Phosphatidylcholine*

PC Molecular Species	% at 2 mo.	% at 10 mo.	% at 26 mo.	2 vs. 10 mo.	2 vs. 26 mo.	10 vs. 26 mo.
**32:00**	8.33	8.78	8.07	$p = 0.0027$	ns	$p < 0.0001$
**32:01**	1.33	1.71	2.03	$p = 0.0142$	$p < 0.0001$	ns
**34:00**	3.74	3.45	2.87	ns	$p < 0.0001$	$p < 0.0001$
**34:01**	36.36	37.85	39.99	$p < 0.0001$	$p < 0.0001$	$p < 0.0001$
**34:02**	1.20	1.19	1.20	ns	ns	ns
**36:01**	16.95	17.30	16.96	$p = 0.0291$	ns	$p = 0.0312$
**36:02**	5.22	5.15	5.14	ns	ns	ns
**36:03**	0.69	0.62	0.61	ns	ns	ns
**36:04**	2.39	2.17	2.36	ns	ns	ns
**38:01**	1.56	1.51	1.44	ns	ns	ns
**38:02**	2.05	2.30	2.25	ns	ns	ns
**38:03**	0.83	1.05	1.01	ns	ns	ns
**38:04**	3.18	2.84	2.82	$p = 0.0339$	$p = 0.0228$	ns
**38:05**	1.11	0.99	1.12	ns	ns	ns
**38:06**	7.58	6.86	6.46	$p < 0.0001$	$p < 0.0001$	$p = 0.0107$
**40:06**	4.31	3.84	3.39	$p = 0.0018$	$p < 0.0001$	$p = 0.0035$
**40:07**	1.57	1.41	1.30	ns	ns	ns
**44:12**		0.43	0.40	ns	ns	ns

Supplementary *Table S11: Molecular Species Composition of Brainstem Phosphatidylethanolamine*

PE Molecular Species	% at 2 mo.	% at 10 mo.	% at 26 mo.	2 vs. 10 mo.	2 vs. 26 mo.	10 vs. 26 mo.
**34:00**	0.19	0.20	0.17	ns	ns	ns
**34:00p**	0.61	0.72	0.91	ns	ns	ns
**34:01**	5.70	6.74	7.20	p < 0.0001	p < 0.0001	p = 0.0029
**34:01p**	0.63	0.64	0.66	ns	ns	ns
**34:02**	0.55	0.60	0.65	ns	ns	ns
**36:00**	0.20	0.23	0.18	ns	ns	ns
**36:00p**	0.73	0.84	1.06	ns	p = 0.0422	ns
**36:01**	8.52	10.49	11.43	p < 0.0001	p < 0.0001	p < 0.0001
**36:01p**	1.27	1.39	1.48	ns	ns	ns
**36:02**	10.67	10.86	11.32	ns	p < 0.0001	p = 0.0026
**36:02p**	1.17	1.55	1.87	p = 0.0163	p < 0.0001	ns
**36:03**	0.66	0.64	0.67	ns	ns	ns
**36:04**	1.88	2.12	2.49	ns	p < 0.0001	p = 0.0227
**38:00**	0.69	0.58	0.48	ns	ns	ns
**38:00p**	0.39	0.40	0.50	ns	ns	ns
**38:01**	1.63	1.76	1.70	ns	ns	ns
**38:01p**	0.77	0.91	0.97	ns	ns	ns
**38:02**	2.41	2.28	2.30	ns	ns	ns
**38:02p**	0.67	0.87	0.98	ns	ns	ns
**38:03**	0.80	0.68	0.72	ns	ns	ns
**38:03p**	0.49	0.42	0.49	ns	ns	ns
**38:04**	10.06	9.90	10.59	ns	p = 0.0004	p < 0.0001
**38:04p**	0.58	0.50	0.55	ns	ns	ns
**38:05**	4.61	4.60	5.31	ns	p < 0.0001	p < 0.0001
**38:05p**	0.67	0.68	0.76	ns	ns	ns
**38:06**	6.64	5.63	4.88	p < 0.0001	p < 0.0001	p < 0.0001
**40:04**	2.23	2.06	1.93	ns	ns	ns
**40:04p**	0.42	0.45	0.44	ns	ns	ns
**40:05**	1.26	1.15	1.19	ns	ns	ns
**40:05p**	0.94	0.84	0.80	ns	ns	ns
**40:06**	27.68	22.76	19.14	p < 0.0001	p < 0.0001	p < 0.0001
**40:07**	2.54	2.34	2.11	ns	p = 0.0053	ns
**44:10**	0.96	0.85	0.70	ns	ns	ns
**44:12**	0.78	0.79	0.66	ns	ns	ns
**Σ Ether PE**	9.33	10.53	11.81	p < 0.0001	p < 0.0001	p < 0.0001

Supplementary *Table S12: Molecular Species Composition of Brainstem Phosphatidylserine*

PS Molecular Species	% at 2 mo.	% at 10 mo.	% at 26 mo.	2 vs. 10 mo.	2 vs. 26 mo.	10 vs. 26 mo.
**34:01**	1.38	1.22	1.12	ns	ns	ns
**36:01**	37.04	45.02	48.76	$p < 0.0001$	$p < 0.0001$	$p < 0.0001$
**36:02**	9.89	9.41	9.06	ns	ns	ns
**38:01**	7.17	7.57	7.44	ns	ns	ns
**38:02**	3.17	2.37	2.36	ns	ns	ns
**38:04**	3.65	3.91	4.49	ns	ns	ns
**38:08**	0.33	0.44	0.64	ns	ns	ns
**40:01**	2.36	1.87	2.12	ns	ns	ns
**40:02**	1.48	1.53	1.51	ns	ns	ns
**40:04**	3.25	3.11	2.92	ns	ns	ns
**40:06**	22.78	16.48	12.69	$p < 0.0001$	$p < 0.0001$	$p < 0.0001$
**40:07**	0.59	0.63	0.52	ns	ns	ns
**42:01**	1.34	1.01	1.22	ns	ns	ns
**42:02**	0.93	0.88	1.09	ns	ns	ns
**42:08**	0.47	0.65	0.63	ns	ns	ns
**42:09**	0.52	0.82	0.74	ns	ns	ns
**44:10**	1.42	1.02	0.74	ns	ns	ns
**44:12**	1.66	1.22	1.00	ns	ns	ns

Supplementary *Table S13: Molecular Species Composition of Cortical Phosphatidylcholine*

PC Molecular Species	% at 2 mo.	% at 10 mo.	% at 26 mo.	2 vs. 10 mo.	2 vs. 26 mo.	10 vs. 26 mo.
**30:00**	0.36	0.32	0.30	ns	ns	ns
**32:00**	22.49	23.54	22.55	$p < 0.0001$	ns	$p < 0.0001$
**32:01**	2.21	2.35	2.40	ns	ns	ns
**32:02**	0.84	0.86	0.87	ns	ns	ns
**34:00**	5.91	5.92	5.51	ns	$p = 0.0269$	$p = 0.0213$
**34:00p**	0.62	0.57	0.57	ns	ns	ns
**34:01**	34.06	33.74	34.38	ns	ns	$p = 0.0001$
**34:02**	1.25	1.25	1.26	ns	ns	ns
**36:01**	7.94	8.14	8.42	ns	$p = 0.0051$	ns
**36:02**	2.48	2.30	2.50	ns	ns	ns
**36:03**	0.75	0.63	0.73	ns	ns	ns
**36:04**	5.25	4.86	4.92	$p = 0.0293$	ns	ns
**38:01**	0.28	0.25	0.25	ns	ns	ns
**38:02**	0.38	0.35	0.38	ns	ns	ns
**38:03**	0.35	0.37	0.35	ns	ns	ns
**38:04**	5.00	4.68	4.55	ns	$p = 0.0092$	ns
**38:05**	1.41	1.27	1.37	ns	ns	ns
**38:06**	4.76	4.84	4.94	ns	ns	ns
**40:04**	0.35	0.34	0.34	ns	ns	ns
**40:06**	2.48	2.51	2.49	ns	ns	ns
**40:07**	0.85	0.91	0.94	ns	ns	ns

Supplementary *Table S14: Molecular Species Composition of Cortical Phosphatidylethanolamine*

PE Molecular Species	% at 2 mo.	% at 10 mo.	% at 26 mo.	2 vs. 10 mo.	2 vs. 26 mo.	10 vs. 26 mo.
**32:00**	0.16	0.12	0.06	ns	ns	ns
**34:00**	0.41	0.44	0.28	ns	ns	ns
**34:00p**	0.42	0.31	0.31	ns	ns	ns
**34:01**	3.28	2.04	2.38	$p < 0.0001$	$p < 0.0001$	ns
**36:00**	0.60	0.62	0.57	ns	ns	ns
**36:01**	3.02	3.47	3.96	ns	$p < 0.0001$	$p = 0.0460$
**36:02**	4.28	4.03	3.77	ns	$p = 0.0415$	ns
**36:03**	0.48	0.38	0.34	ns	ns	ns
**36:04**	2.53	1.85	1.79	$p = 0.0013$	$p = 0.0013$	ns
**38:00**	1.08	1.26	1.00	ns	ns	ns
**38:01**	0.44	0.45	0.50	ns	ns	ns
**38:02**	0.23	0.16	0.34	ns	ns	ns
**38:03**	0.65	0.94	0.65	ns	ns	ns
**38:03p**	0.55	0.56	0.57	ns	ns	ns
**38:04**	19.01	17.80	17.33	$p < 0.0001$	$p < 0.0001$	ns
**38:05**	2.72	2.43	3.21	ns	ns	$p = 0.0006$
**38:05p**	0.87	0.91	0.97	ns	ns	ns
**38:06**	12.49	12.94	13.12	ns	$p = 0.0077$	ns
**40:04**	3.01	3.10	2.69	ns	ns	ns
**40:05p**	0.84	1.03	0.89	ns	ns	ns
**40:06**	37.38	37.31	37.78	ns	ns	ns
**40:07**	3.04	3.18	3.01	ns	ns	ns
**40:08**	0.22	0.22	0.15	ns	ns	ns
**44:10**	0.28	0.21	0.17	ns	ns	ns
**Σ Ether PE**	2.68	3.35	3.26	$p = 0.0016$	$p = 0.0152$	ns

Supplementary *Table S15: Molecular Species Composition of Cortical Phosphatidylserine*

PS Molecular Species	% at 2 mo.	% at 10 mo.	% at 26 mo.	2 vs. 10 mo.	2 vs. 26 mo.	10 vs. 26 mo.
**34:01**	1.30	0.85	1.22	ns	ns	ns
**36:00**	0.86	0.83	0.70	ns	ns	ns
**36:01**	6.41	7.37	8.90	ns	p = 0.0366	ns
**36:02**	4.94	3.19	3.47	ns	ns	ns
**38:01**	0.40	0.20	0.67	ns	ns	ns
**38:04**	2.33	1.81	1.58	ns	ns	ns
**38:06**	0.98	0.17	0.20	ns	ns	ns
**40:01**	0.49	0.43	1.24	ns	ns	ns
**40:04**	3.48	4.26	6.15	ns	p = 0.0231	ns
**40:05**	1.80	2.97	0.98	ns	ns	ns
**40:06**	74.57	72.24	68.92	ns	p < 0.0001	p = 0.0035
**40:07**	1.49	0.82	1.25	ns	ns	ns
**44:10**	0.96	0.57	0.84	ns	ns	ns

Supplementary *Table S16: Molecular Species Composition of White Matter Phosphatidylcholine*

PC Molecular Species	% at 2 mo.	% at 10 mo.	% at 26 mo.	2 vs. 10 mo.	2 vs. 26 mo.	10 vs. 26 mo.
**30:00**	0.46	0.41	0.46	ns	ns	ns
**32:00**	10.38	13.16	9.28	$p < 0.0001$	$p = 0.0064$	$p < 0.0001$
**32:01**	1.71	2.00	2.34	ns	ns	ns
**32:02**	0.30	0.46	0.53	ns	ns	ns
**34:00**	3.04	2.63	1.78	ns	$p = 0.0015$	$p = 0.0475$
**34:00p**	0.96	0.73	0.86	ns	ns	ns
**34:01**	32.23	33.74	35.84	$p < 0.0001$	$p < 0.0001$	$p < 0.0001$
**34:02**	1.18	1.13	1.13	ns	ns	ns
**36:00p**	0.68	0.56	0.79	ns	ns	ns
**36:01**	20.55	21.72	22.98	$p = 0.0013$	$p < 0.0001$	$p = 0.0015$
**36:02**	4.82	4.51	4.78	ns	ns	ns
**36:03**	0.67	0.56	0.51	ns	ns	ns
**36:04**	4.00	3.68	3.85	ns	ns	ns
**38:01**	1.53	1.18	1.31	ns	ns	ns
**38:02**	1.39	1.33	1.52	ns	ns	ns
**38:03**	0.68	0.62	0.72	ns	ns	ns
**38:04**	5.04	4.62	4.76	ns	ns	ns
**38:05**	1.48	1.32	1.33	ns	ns	ns
**38:06**	3.12	2.68	2.26	ns	$p = 0.0446$	ns
**40:01**	0.63	0.26	0.49	ns	ns	ns
**40:02**	0.48	0.36	0.43	ns	ns	ns
**40:04**	0.41	0.38	0.41	ns	ns	ns
**40:06**	1.62	1.46	1.24	ns	ns	ns
**40:07**	0.61	0.53	0.40	ns	ns	ns

Supplementary *Table S17: Molecular Species Composition of White Matter Phosphatidylethanolamine*

PE Molecular Species	% at 2 mo.	% at 10 mo.	% at 26 mo.	2 vs. 10 mo.	2 vs. 26 mo.	10 vs. 26 mo.
**34:00**	0.15	0.17	0.12	ns	ns	ns
**34:00p**	0.78	0.80	0.99	ns	ns	ns
**34:01**	4.58	5.81	5.73	p = 0.0047	p = 0.0173	ns
**34:01p**	0.70	0.34	0.49	ns	ns	ns
**34:02**	0.52	0.47	0.49	ns	ns	ns
**36:00**	0.31	0.25	0.18	ns	ns	ns
**36:00p**	0.65	0.77	1.03	ns	ns	ns
**36:01**	11.89	14.63	15.62	p < 0.0001	p < 0.0001	p = 0.0488
**36:01p**	1.39	1.37	1.53	ns	ns	ns
**36:02**	7.29	7.60	8.05	ns	ns	ns
**36:02p**	1.52	1.88	2.31	ns	ns	ns
**36:03**	0.55	0.53	0.53	ns	ns	ns
**36:04**	2.15	2.30	2.80	ns	ns	ns
**38:00**	0.86	0.59	0.60	ns	ns	ns
**38:00p**	0.36	0.38	0.51	ns	ns	ns
**38:01**	1.93	1.72	1.85	ns	ns	ns
**38:01p**	0.73	0.78	0.88	ns	ns	ns
**38:02**	2.19	1.91	2.02	ns	ns	ns
**38:02p**	0.66	0.83	1.14	ns	ns	ns
**38:03**	1.11	0.96	0.99	ns	ns	ns
**38:03p**	0.71	0.66	0.73	ns	ns	ns
**38:04**	17.50	15.85	17.04	p < 0.0001	ns	p = 0.0126
**38:04p**	0.95	0.69	0.68	ns	ns	ns
**38:05**	4.30	3.65	4.49	ns	ns	ns
**38:05p**	1.19	1.12	1.22	ns	ns	ns
**38:06**	5.92	5.24	3.93	ns	p < 0.0001	p = 0.0055
**40:01**	0.17	0.14	0.19	ns	ns	ns
**40:02**	0.36	0.25	0.35	ns	ns	ns
**40:03p**	0.37	0.31	0.38	ns	ns	ns
**40:04**	3.67	3.95	4.02	ns	ns	ns
**40:04p**	0.64	0.53	0.61	ns	ns	ns
**40:05**	1.83	1.54	1.74	ns	ns	ns
**40:05p**	1.23	1.06	1.15	ns	ns	ns
**40:06**	17.64	15.77	10.53	p < 0.0001	p < 0.0001	p < 0.0001
**40:07**	2.50	2.14	1.79	ns	ns	ns
**40:08**	0.21	0.19	0.23	ns	ns	ns
**40:09**	0.16	0.19	0.17	ns	ns	ns
**44:10**	0.32	0.23	0.21	ns	ns	ns
**Σ Ether PE**	11.88	11.73	13.89	ns	p < 0.0001	p < 0.0001

Supplementary *Table S18: Molecular Species Composition of White Matter Phosphatidylserine*

PS Molecular Species	% at 2 mo.	% at 10 mo.	% at 26 mo.	2 vs. 10 mo.	2 vs. 26 mo.	10 vs. 26 mo.
**34:01**	0.70	0.46	0.57	ns	ns	ns
**36:00**	2.41	1.00	0.95	ns	ns	ns
**36:01**	35.99	48.15	52.69	p < 0.0001	p < 0.0001	p < 0.0001
**36:02**	4.87	3.82	3.99	ns	ns	ns
**38:01**	5.12	4.64	5.05	ns	ns	ns
**38:02**	2.24	1.86	1.91	ns	ns	ns
**38:04**	5.87	5.67	5.94	ns	ns	ns
**40:01**	2.06	1.22	2.26	ns	ns	ns
**40:02**	1.81	1.77	1.56	ns	ns	ns
**40:04**	4.93	5.23	5.04	ns	ns	ns
**40:05**	1.09	0.31	0.53	ns	ns	ns
**40:06**	26.67	21.37	13.65	p < 0.0001	p < 0.0001	p < 0.0001
**40:07**	0.84	0.76	0.50	ns	ns	ns
**40:08**	0.38	0.52	0.68	ns	ns	ns
**42:01**	0.97	0.47	0.98	ns	ns	ns
**42:02**	1.06	0.69	1.12	ns	ns	ns
**42:08**	0.54	0.35	0.66	ns	ns	ns
**42:09**	0.57	1.02	0.86	ns	ns	ns

Supplementary *Table S19: Molecular Species Composition of Midbrain Phosphatidylcholine*

**PC Molecular Species**	**% at 2 mo.**	**% at 10 mo.**	**% at 26 mo.**	**2 vs. 10 mo.**	**2 vs. 26 mo.**	**10 vs. 26 mo.**
**32:00**	13.84	13.38	12.78	p = 0.0232	p < 0.0001	p = 0.0022
**32:01**	1.71	1.90	2.09	ns	ns	ns
**34:00**	4.70	4.15	3.67	p = 0.0051	p < 0.0001	p = 0.0162
**34:01**	36.52	37.40	37.59	p < 0.0001	p < 0.0001	ns
**34:02**	1.22	1.25	1.26	ns	ns	ns
**36:01**	13.92	14.35	14.13	p = 0.0367	ns	ns
**36:02**	4.03	4.11	4.38	ns	ns	ns
**36:03**	0.69	0.69	0.69	ns	ns	ns
**36:04**	4.15	3.90	4.29	ns	ns	ns
**38:01**	0.78	0.67	0.63	ns	ns	ns
**38:02**	0.86	0.93	0.94	ns	ns	ns
**38:04**	5.09	4.72	4.71	ns	ns	ns
**38:05**	1.59	1.48	1.61	ns	ns	ns
**38:06**	5.95	6.20	6.34	ns	ns	ns
**40:06**	3.58	3.52	3.47	ns	ns	ns
**40:07**	1.38	1.38	1.43	ns	ns	ns

Supplementary *Table S20: Molecular Species Composition of Midbrain Phosphatidylethanolamine*

PE Molecular Species	% at 2 mo.	% at 10 mo.	% at 26 mo.	2 vs. 10 mo.	2 vs. 26 mo.	10 vs. 26 mo.
**34:00**	0.25	0.21	0.18	ns	ns	ns
**34:00p**	0.47	0.58	0.69	ns	ns	ns
**34:01**	4.68	4.93	5.20	ns	$p = 0.0026$	ns
**34:01p**	0.28	0.27	0.32	ns	ns	ns
**34:02**	0.50	0.50	0.56	ns	ns	ns
**36:00**	0.35	0.31	0.26	ns	ns	ns
**36:00p**	0.32	0.45	0.38	ns	ns	ns
**36:01**	6.52	7.46	8.02	$p < 0.0001$	$p < 0.0001$	$p = 0.0011$
**36:01p**	0.66	0.76	0.74	ns	ns	ns
**36:02**	7.79	8.30	8.59	$p = 0.0027$	$p < 0.0001$	ns
**36:02p**	0.68	1.01	1.21	ns	$p = 0.0019$	ns
**36:03**	0.53	0.53	0.56	ns	ns	ns
**36:04**	2.15	2.18	2.58	ns	$p = 0.0148$	$p = 0.0280$
**38:00**	0.93	0.92	0.98	ns	ns	ns
**38:00p**	0.20	0.14	0.21	ns	ns	ns
**38:01**	0.91	0.78	0.73	ns	ns	ns
**38:01p**	0.28	0.37	0.37	ns	ns	ns
**38:02**	0.95	0.93	0.98	ns	ns	ns
**38:02p**	0.33	0.39	0.55	ns	ns	ns
**38:03**	0.81	0.67	0.68	ns	ns	ns
**38:03p**	0.56	0.58	0.59	ns	ns	ns
**38:04**	14.70	14.22	14.61	$p = 0.0049$	ns	$p = 0.0293$
**38:04p**	0.64	0.59	0.61	ns	ns	ns
**38:05**	3.86	4.03	4.80	ns	$p < 0.0001$	$p < 0.0001$
**38:05p**	0.92	1.00	1.17	ns	ns	ns
**38:06**	8.09	7.91	7.44	ns	$p < 0.0001$	$p = 0.0064$
**40:03p**	0.43	0.42	0.40	ns	ns	ns
**40:04**	3.00	2.93	2.78	ns	ns	ns
**40:04p**	0.42	0.43	0.51	ns	ns	ns
**40:05**	1.18	1.04	1.00	ns	ns	ns
**40:05p**	1.20	1.19	1.12	ns	ns	ns
**40:06**	31.33	29.81	27.16	$p < 0.0001$	$p < 0.0001$	$p < 0.0001$
**40:07**	2.90	2.94	2.80	ns	ns	ns
**44:10**	0.76	0.76	0.75	ns	ns	ns
**44:12**	0.46	0.49	0.50	ns	ns	ns
**Σ Ether PE**	7.37	8.16	8.86	$p < 0.0001$	$p < 0.0001$	$p < 0.0001$

Supplementary *Table S21: Molecular Species Composition of Midbrain Phosphatidylserine*

PS Molecular Species	% at 2 mo.	% at 10 mo.	% at 26 mo.	2 vs. 10 mo.	2 vs. 26 mo.	10 vs. 26 mo.
**34:01**	1.18	1.20	0.65	ns	ns	ns
**36:00**	1.39	1.11	1.06	ns	ns	ns
**36:01**	22.13	28.85	31.99	$p < 0.0001$	$p < 0.0001$	$p < 0.0001$
**36:02**	8.09	7.30	7.43	ns	ns	ns
**38:01**	2.70	2.43	2.44	ns	ns	ns
**38:02**	1.26	1.31	1.18	ns	ns	ns
**38:04**	3.58	4.34	5.35	ns	$p = 0.0018$	ns
**38:05**	0.61	0.62	0.66	ns	ns	ns
**40:01**	1.22	1.04	1.50	ns	ns	ns
**40:02**	0.88	1.13	1.12	ns	ns	ns
**40:04**	4.08	4.16	4.25	ns	ns	ns
**40:05**	0.67	1.07	1.39	ns	ns	ns
**40:06**	46.52	39.39	35.41	$p < 0.0001$	$p < 0.0001$	$p < 0.0001$
**40:07**	1.25	0.93	1.01	ns	ns	ns
**42:01**	0.53	0.40	0.62	ns	ns	ns
**42:02**	0.54	0.36	0.72	ns	ns	ns
**42:09**	0.71	0.81	0.73	ns	ns	ns
**44:10**	1.47	1.00	1.03	ns	ns	ns
**44:12**	1.15	0.80	1.02	ns	ns	ns

Supplementary *Table S22: Total nanomoles lipid phosphorus per mg wet weight of tissue, compared within regions at each age.*

nmol. P/mg.	2 months	10 months	26 months	2 vs. 10 mo.	2 vs. 26 mo.	10 vs 26 mo.
**Retina**	36.9	31.2	35.3	ns	ns	ns
**Cortex**	39.4	39.1	37.3	ns	ns	ns
**Hippocampus**	49.4	44.6	52.9	ns	ns	ns
**Cerebellum**	40.3	41.2	44.1	ns	ns	ns
**Midbrain**	38.5	38.3	36.7	ns	ns	ns
**Brainstem**	58.4	55.4	60.3	ns	ns	ns
**White Matter**	49.2	58.2	65	ns	p = 0.0025	ns

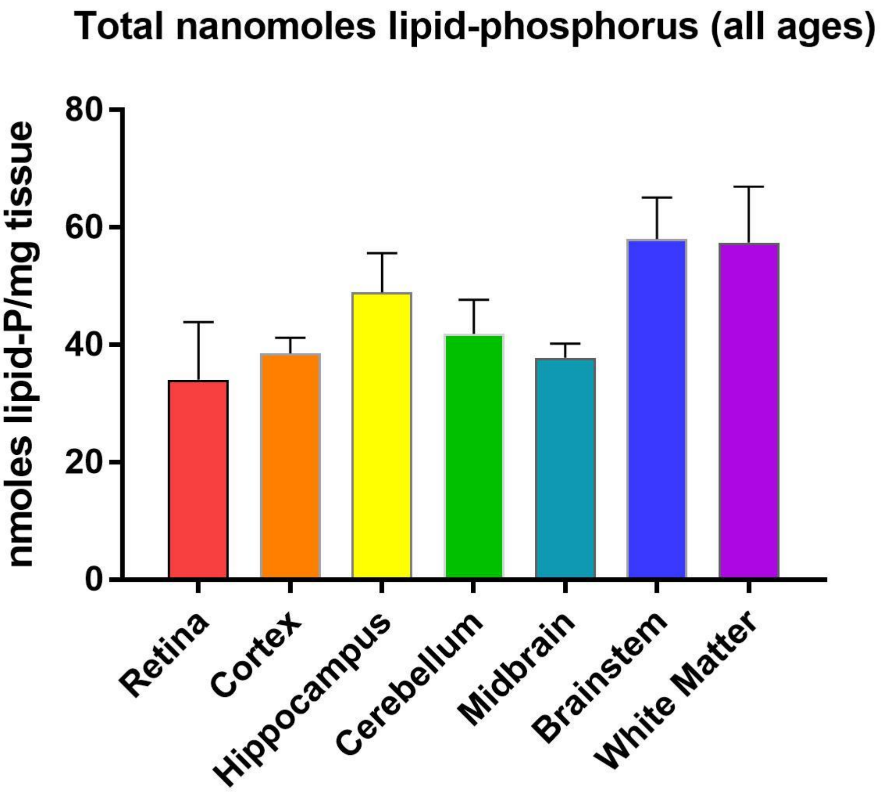

Supplementary Figure S1: Total nanomoles lipid-phosphorus per mg wet weight of tissue, combined ages for each region. Total lipid phosphorus was not significantly different within each region at the different ages with the exception of white matter, which was only weakly significant ($p = 0.0468$). Therefore all lipid-phosphorus data were collapsed to give an $n = 12$ (except for retina, where $n = 8$). We then compared the difference in total lipid-phosphorus between regions. Statistics: Raw data was transformed to $\log_{10}$ [Y = log(Y)] to regain a normal distribution and One-way ANOVA with Tukey's Multiple Comparisons Test was appropriately used (see supplementary table 23 for statistical comparison of the above means).

Supplementary *Table S23: Total nanomoles lipid-phosphorus per mg wet weight of tissue, compared between regions at all ages combined.*

**Tissue (all ages combined)**							
**Retina (34.08 nmoles/mg**							
**Cortex (38.58 nmoles/mg)**	ns						
**Hippocampus (48.97 nmoles/mg)**	p < 0.0001	p = 0.003					
**Cerebellum (41.91 nmoles/mg)**	p = 0.0127	ns	ns				
**Midbrain (37.84 nmoles/mg)**	ns	ns	p = 0.001	ns			
**Brainstem (58.07 nmoles/mg)**	p < 0.0001	p < 0.0001	ns	p < 0.001	p < 0.0001		
**White Matter (57.42 nmoles/mg)**	p < 0.0001	p < 0.0001	ns	p < 0.001	p < 0.0001	ns	
**Values below shaded chart area indicate the significance (p-value) of each inter-tissue comparison.**	**Retina**	**Cortex**	**Hippocampus**	**Cerebellum**	**Midbrain**	**Brainstem**	**White Matter**

Supplementary Table S24: nmoles PC per mg wet weight (top) & percent PC relative to PE & PS (below), compared within regions at each age.

**nmol. PC**	**2 months**	**10 months**	**26 months**	**2 vs. 10 mo.**	**2 vs. 26 mo.**	**10 vs 26 mo.**
**Retina**	12.9	15.4	14.6	ns	ns	ns
**Cortex**	16.4	20	15.3	p = 0.0255	ns	p = 0.0029
**Hippocampus**	9.8	11.1	16.9	ns	p < 0.0001	p = 0.0001
**Cerebellum***	20.9	-	22.7	-	ns	-
**Midbrain**	19.8	18.2	19.3	ns	ns	ns
**Brainstem**	18.7	19.4	19.4	ns	ns	ns
**White Matter**	14.2	13.7	14.5	ns	ns	ns
**% PC**	**2 months**	**10 months**	**26 months**	**2 vs. 10 mo.**	**2 vs. 26 mo.**	**10 vs 26 mo.**
**Retina**	60.8%	60.5%	58.3%	ns	ns	ns
**Cortex**	78.2%	70.7%	64.3%	ns	p = 0.0121	ns
**Hippocampus**	58.7%	56.8%	58.3%	ns	ns	ns
**Cerebellum**	67.2%	62.4%	64.2%	ns	ns	ns
**Midbrain**	56.8%	54.8%	52.8%	ns	ns	ns
**Brainstem**	56.4%	54.2%	52.1%	ns	p = 0.0158	ns
**White Matter**	40.7%	34.5%	33.9%	p < 0.0001	p < 0.0001	ns

** Wet weight could not be accurately determined for 10 month cerebellum samples.*

Supplementary *Table S25: nmoles PE per mg wet weight (top) & percent PE relative to PC & PS (below), compared within regions at each age.*

**nmol. PE**	**2 months**	**10 months**	**26 months**	**2 vs. 10 mo.**	**2 vs. 26 mo.**	**10 vs 26 mo.**
**Retina**	6.8	8.3	8.2	ns	ns	ns
**Cortex**	4.3	7.6	7.3	p = 0.0435	ns	ns
**Hippocampus**	6.1	6.9	7.2	ns	ns	ns
**Cerebellum***	8.7	-	10.7	-	ns	-
**Midbrain**	13.4	13.2	15.3	ns	ns	ns
**Brainstem**	12.4	13.9	14.9	ns	p= 0.0228	ns
**White Matter**	11.6	14.8	16.3	p = 0.0402	p = 0.0021	ns
**% PE**	**2 months**	**10 months**	**26 months**	**2 vs. 10 mo.**	**2 vs. 26 mo.**	**10 vs 26 mo.**
**Retina**	31.7%	32.8%	32.9%	ns	ns	ns
**Cortex**	19.1%	26.3%	32.6%	ns	p = 0.0149	ns
**Hippocampus**	35.8%	35.7%	36.6%	ns	ns	ns
**Cerebellum**	27.9%	31.6%	29.0%	ns	ns	ns
**Midbrain**	38.4%	39.4%	41.8%	ns	ns	ns
**Brainstem**	37.2%	38.9%	39.9%	ns	ns	ns
**White Matter**	33.1%	37.5%	38.3%	p = 0.0027	p = 0.0005	ns

** Wet weight could not be accurately determined for 10 month cerebellum samples.*

Supplementary *Table S26: nmoles PS per mg wet weight (top) & percent PS relative to PC & PE (below), compared within regions at each age.*

**nmol. PS**	**2 months**	**10 months**	**26 months**	**2 vs. 10 mo.**	**2 vs. 26 mo.**	**10 vs 26 mo.**
**Retina**	1.6	1.7	2.2	ns	ns	ns
**Cortex**	0.6	0.8	0.7	ns	ns	ns
**Hippocampus**	0.9	1.5	1.5	ns	ns	ns
**Cerebellum***	1.5	-	2.5	-	ns	-
**Midbrain**	1.7	1.9	2	ns	ns	ns
**Brainstem**	2.1	2.5	3	ns	ns	ns
**White Matter**	9.2	11.1	11.9	ns	ns	ns
**% PS**	**2 months**	**10 months**	**26 months**	**2 vs. 10 mo.**	**2 vs. 26 mo.**	**10 vs 26 mo.**
**Retina**	7.5%	6.6%	8.8%	ns	ns	ns
**Cortex**	2.7%	3.0%	3.1%	ns	ns	ns
**Hippocampus**	5.5%	7.5%	5.1%	ns	ns	ns
**Cerebellum**	4.8%	6.1%	6.8%	ns	ns	ns
**Midbrain**	4.8%	5.8%	5.4%	ns	ns	ns
**Brainstem**	6.4%	6.9%	8.1%	ns	ns	ns
**White Matter**	26.3%	28.0%	27.8%	ns	ns	ns

** Wet weight could not be accurately determined for 10 month cerebellum samples.*
